# Blood Parasite Infections and Reproductive Performance in a Multi-Host Avian System

**DOI:** 10.3390/biology15181583

**Published:** 2026-09-09

**Authors:** Karolina Żukowska, Lars Gustafsson, Edyta Podmokła

**Affiliations:** 1 Department of Comparative Anatomy, Institute of Zoology and Biomedical Research, Faculty of Biology, Jagiellonian University, Gronostajowa 9, 30-387 Kraków, Poland; 2 Department of Ecology and Genetics, Evolutionary Biology Centre, Uppsala University, Norbyvägen 18 D, SE-752 36 Uppsala, Sweden

**Keywords:** avian malaria, blood parasites, breeding success, *Haemoproteus*, host–parasite interactions, parasite intensity, reproductive performance, *Trypanosoma*

## Abstract

Blood parasites are widespread pathogens of wild birds, yet their reproductive consequences remain poorly understood. We investigated associations between blood parasite infections and reproductive performance in three passerine species breeding sympatrically on Gotland, Sweden: the resident great tit (*Parus major*) and blue tit (*Cyanistes caeruleus*) and the migratory collared flycatcher (*Ficedula albicollis*). The study was based on data from 181 nests and 1201 nestling observations. Adult birds were screened for *Plasmodium*, *Haemoproteus*, and *Trypanosoma* infections using next-generation sequencing, and infection intensity of malaria parasites (*Plasmodium/Haemoproteus*) and *Trypanosoma* was quantified using quantitative PCR. We tested whether parental infection status and parasite intensity were associated with reproductive traits measured at the nest level (hatching date, clutch size, and brood size) and nestling level (body mass, growth, and condition). Associations between infections and reproduction differed among parasite groups, parental sexes, and host species, with some of the strongest and most consistent patterns involving *Haemoproteus* infections. Most significant relationships were detected for nestling traits rather than breeding parameters, and a substantial proportion of associations remained significant after false discovery rate (FDR) correction. Both positive and negative associations were observed, and significant interactions between female and male infection status indicated that offspring performance depended on both parents’ infection status. Moreover, analyses based on infection status and parasite intensity revealed partly different patterns of association with reproductive traits. Overall, these results demonstrate that the reproductive consequences of blood parasite infections are parasite-specific and context-dependent.

## Simple Summary

Parasites are an important part of natural ecosystems and can affect the health and survival of wild animals. Among them are blood parasites, which infect millions of birds worldwide. However, their effects on reproduction remain poorly understood. We investigated whether blood parasite infections were associated with breeding performance in three bird species nesting in the same environment in Sweden: great tits, blue tits, and collared flycatchers. We examined whether infected parents differed from uninfected ones in the number and quality of offspring produced. The relationships between infections and reproduction varied among parasite groups, bird species, and parental sex. Most associations were detected for offspring growth and body condition rather than for the timing of breeding or the number of eggs and nestlings. While some infections were associated with poorer offspring performance, others showed neutral or positive relationships. We also found that offspring performance often depended on the infection status of both parents. These results demonstrate that the reproductive consequences of blood parasite infections are complex and context-dependent. Understanding these relationships is important for explaining how parasites shape the ecology and evolution of wild bird populations.

## 1. Introduction

Reproduction is one of the most energetically demanding stages of an organism’s life. Because resources available to individuals are finite, investment in current reproduction must often be balanced against other fitness-related functions, including growth, maintenance, survival, and immune defense. Life-history theory predicts that such allocation decisions generate trade-offs, whereby increased investment in one function is accompanied by reduced investment in another [1]. Parasites represent an additional source of physiological costs because they exploit host resources, damage tissues, stimulate immune responses, and alter host physiology and behavior [2,3]. Consequently, infections may affect not only host condition and survival, but also reproductive investment and offspring performance. Consistent with this prediction, a comparative meta-analysis showed that parasitism generally reduces host reproductive fitness, including fecundity, offspring viability, and mating success [4].

Avian blood parasites constitute one of the most widespread and diverse groups of parasites infecting wild birds. They include intracellular haemosporidians of the genera *Plasmodium* and *Haemoproteus*, as well as extracellular flagellates of the genus *Trypanosoma* [5]. These parasites are transmitted by blood-feeding dipteran vectors but differ substantially in their life cycles, transmission routes, tissue tropism, and pathogenicity [6,7,8]. Whereas *Plasmodium* and *Haemoproteus* undergo extensive exoerythrocytic development within host tissues before infecting erythrocytes, avian trypanosomes occur primarily as extracellular blood parasites. Consequently, infections caused by different parasite groups may vary considerably in their effects on host condition, behavior, survival, and reproductive performance [2,7].

Despite their potential to influence host fitness, studies investigating the reproductive consequences of these parasites have produced inconsistent results. Haemosporidian infections have been associated with reduced reproductive performance in some systems, including lower breeding success, reduced offspring quality, and decreased reproductive output [9,10]. Experimental reduction in chronic malaria infections has likewise been shown to increase hatching success, parental provisioning rates, and fledging success, providing direct evidence that blood parasites can impose reproductive costs [11,12]. However, other studies have reported weak or no associations between infection and reproductive traits [13,14], while some have found positive relationships between infection and reproductive success or reproductive effort [15,16]. For example, a long-term study of great tits (*Parus major* Linnaeus, 1758) showed that haemosporidian infection and co-infection were associated with increased reproductive success despite reduced survival of co-infected individuals [17]. Studies examining avian *Trypanosoma* infections have linked them to reduced offspring growth [18], altered parental behavior [19,20], or lower offspring immune responsiveness [21], but evidence for direct effects on overall reproductive success remains limited and inconsistent. Overall, syntheses of available evidence indicate that blood parasites can affect multiple components of host reproductive fitness, although the strength and direction of these associations differ considerably among host–parasite systems [4,22].

Several factors may contribute to this variability. Avian blood parasites differ markedly in their biology, virulence, and pathogenicity, and even closely related parasite lineages may have contrasting effects on host fitness [23,24]. Moreover, few correlates of haemosporidian infections have been consistently demonstrated across studies, suggesting strong effects of host species, parasite lineage, and environmental context [25]. Many avian blood parasites establish long-lasting chronic infections that may persist throughout much of the host’s lifetime [6]. Consequently, infection status may not accurately reflect current infection severity, and individuals carrying chronic low-intensity infections may experience substantially different physiological costs than those with high parasite burdens. This distinction appears biologically important because parasite intensity is often more strongly associated with fitness-related traits than infection prevalence alone [14,26]. The reproductive consequences of infection may also depend on the sex of the infected parent. Female and male birds often differ in exposure and susceptibility to parasitic infections [27,28]. Consequently, infections may affect different components of reproductive performance and impose different fitness costs in females and males [29]. Experimental and observational studies further indicate that parental infections can influence reproductive investment, parental behavior, and offspring performance [12]. In species with biparental care, these effects may additionally depend on the infection or reproductive state of the partner [30,31]. Analyzing infection separately for female and male parents, while also considering their joint effects, may therefore reveal biologically meaningful patterns that remain obscured when infection is assessed only at the level of breeding pairs.

Despite extensive research on avian blood parasites, their consequences on bird’s reproduction traits appear highly context-dependent, with studies reporting negative, neutral, and even positive associations between infection and reproductive performance. Moreover, relatively few studies have simultaneously considered multiple parasite taxa, host species, parental sexes, and both infection status and parasite intensity within a single study system. In this study, we examined whether parental blood parasite infections were associated with reproductive performance in cavity-nesting passerines breeding sympatrically in Gotland, Sweden: the resident great tit and blue tit (*Cyanistes caeruleus* Linnaeus, 1758) and the migratory collared flycatcher (*Ficedula albicollis* Temminck, 1815). Comparing co-occurring resident and migratory species provides an opportunity to assess whether associations between parasite infections and reproduction are consistent across hosts that differ in annual exposure to parasites and life-history strategies. We assessed infections with *Plasmodium*, *Haemoproteus*, and *Trypanosoma* using next-generation sequencing (NGS) and quantified parasite load for combined *Plasmodium/Haemoproteus* and *Trypanosoma* infections using quantitative PCR. Specifically, we tested whether infection status of female and male parents was associated with nest-level reproductive traits, including hatching date, clutch size, and brood size, and nestling-level traits, including body mass, growth, and condition. In addition, we tested whether parasite load among infected females and males was associated with the same reproductive traits. We predicted that if blood parasite infections impose reproductive costs infected parents and/or parents with higher parasite loads would show reduced reproductive performance. However, given previous evidence for context-dependent effects of blood parasite infections, we expected these associations to differ among parasite groups, parental sexes, and host species.

## 2. Materials and Methods

### 2.1. Study Area and Bird Sampling

The study was conducted on populations of three passerine species, the great tit, blue tit, and collared flycatcher, breeding in the southern part of Gotland, Sweden (57°03′ N, 18°17′ E). Field data were collected during the 2019 and 2021 breeding seasons as part of a larger research project investigating host–parasite interactions [32]. The 2020 breeding season was not included because fieldwork was suspended due to travel restrictions associated with the COVID-19 pandemic.

**Figure 1:**
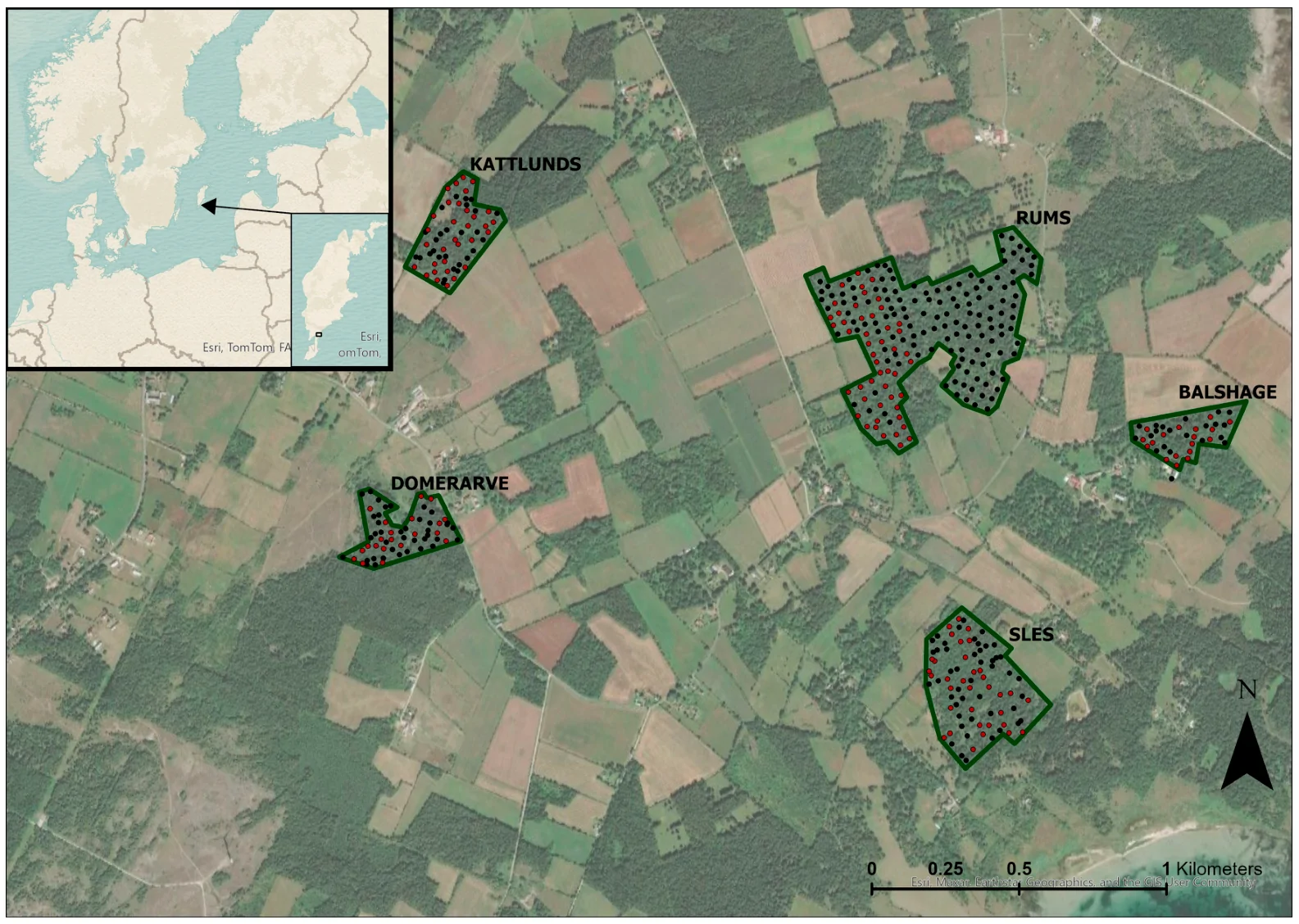
Study area in southern Gotland, Sweden, showing the locations of the five study plots and nest boxes. Red dots indicate nest boxes included in the present study, for which complete breeding information was available for both parental individuals and offspring. Black dots indicate nest boxes that were monitored but not included in the current analyses.

Birds were monitored in five study plots (Figure 1) comprising a total of 424 nest boxes. For the present study, we included only nests for which complete breeding data were available, including information on both parental individuals and offspring. The final dataset consisted of 181 nests (87 great tit, 18 blue tit, and 76 collared flycatcher nests), corresponding to 1201 nestling observations (631, 155, and 415 nestlings, respectively). Numbers of infected individuals and sample sizes available for infection-intensity analyses are provided in Supplementary Table S1.

Nest boxes were inspected regularly throughout the breeding season to record breeding parameters, including hatching date, clutch size, and brood size. Nestlings were ringed with metal bands and weighed on day 8 after hatching. A second measurement was taken near fledging age, i.e., on day 14 in tits and day 12 in collared flycatchers, when nestlings were weighed again and their tarsus length was measured. Adult birds were captured during the nestling–rearing period using nest-box traps or mist nets placed near occupied nests. Blood samples were collected from the brachial vein and stored in 96% ethanol at room temperature until further laboratory analyses. General field procedures, including nest-box monitoring, adult capture, biometric measurements of adult birds, and blood sampling, are described in detail in a previous study from the same system [32].

### 2.2. Molecular Analyses

Genomic DNA was extracted from blood samples using the ammonium acetate precipitation method [33]. DNA concentration was measured using a NanoDrop spectrophotometer (Thermo Fisher Scientific, Waltham, MA, USA), and all samples were diluted to a working concentration of 25 ng/μL prior to molecular analyses.

#### 2.2.1. Blood Parasite Screening: Infection Status

Samples were screened for the presence of *Plasmodium*, *Haemoproteus*, and *Trypanosoma* parasites using high-throughput amplicon sequencing. Parasite-specific markers were amplified in a one-step PCR using tagged primers targeting the mitochondrial cytochrome *b* gene for *Plasmodium* and *Haemoproteus*, and the small subunit ribosomal RNA (SSU rRNA) gene for *Trypanosoma*. To minimize amplification bias, each sample was amplified in duplicate. PCR products were pooled and purified prior to library preparation. Indexed libraries were constructed using a PCR-free protocol and sequenced on an Illumina MiSeq platform (Illumina, San Diego, CA, USA; MiSeq Reagent Kit v2, 500 cycles). Raw sequence data were subjected to quality filtering, demultiplexing, dereplication, and denoising using a standardized bioinformatic pipeline. Amplicon sequence variants were assigned taxonomically through BLAST searches against a custom reference database. To minimize false-positive detections, only parasite lineages represented by more than 10 sequence reads per individual were considered present in subsequent analyses. Haemosporidian lineages were assigned according to the MalAvi database nomenclature [34]. A local copy of the MalAvi database was downloaded from the original MalAvi website (https://wimanet-science.github.io/web/malavi/) on 1 February 2024 and used to construct the reference database for taxonomic assignment. Multiple *Haemoproteus* and *Plasmodium* lineages were detected across the three host species analyzed in this study. A complete list of detected lineages and their frequencies in each host species is provided in Supplementary Table S2. Detailed laboratory procedures, primer sequences, sequencing protocols, and bioinformatic analyses are provided in Podmokła et al. [32].

#### 2.2.2. Blood Parasite Screening: Infection Intensity

Infection intensity was quantified using absolute quantitative PCR (qPCR). Only samples identified as infected during the initial high-throughput sequencing screening were analyzed for the corresponding parasite group. For *Plasmodium* and *Haemoproteus*, qPCR-derived parasite load was estimated using the primer pair described by Fallon et al. [35]. This assay detects both genera but does not discriminate between them. Consequently, qPCR estimates were interpreted as combined *Plasmodium*/*Haemoproteus* parasite load. *Leucocytozoon* infections were not assessed in this study, as the initial high-throughput sequencing screening targeted *Plasmodium* and *Haemoproteus* only. For *Trypanosoma*, infection intensity was quantified using a separate qPCR assay with primers designed for this study based on avian *Trypanosoma* sequences published by Zídková et al. [8].

Standard curves were generated from purified PCR products obtained from samples previously identified as infected with *Plasmodium relictum* (lineage SGS1) and *Trypanosoma culicavium*, respectively. Standard fragments were amplified using primers 292F and 631R for the *Plasmodium*/*Haemoproteus* assay and primers T1617F and T2072R for the *Trypanosoma* assay. Primer sequences, expected product lengths, and their use in qPCR assays or standard curve preparation are provided in Table 1.

Purified PCR products were quantified and serially diluted to construct standard curves spanning a broad range of parasite copy numbers. All qPCR reactions were performed on a QuantStudio 5 Real-Time PCR System (Applied Biosystems, Waltham, MA, USA) using Power SYBR Green PCR Master Mix (Thermo Fisher Scientific, Waltham, MA, USA). Each 25-μL reaction contained 2 μL of template DNA standardized to a concentration of 25 ng/μL (50 ng total DNA per reaction), 12.5 μL of SYBR Green Master Mix, 0.2 μM of each primer, and nuclease-free water. The qPCR assays used primers 343F and 496R for combined *Plasmodium*/*Haemoproteus* parasite load and primers T1871F and T2042R for *Trypanosoma* parasite load (Table 1).

Reactions targeting *Trypanosoma* consisted of an initial denaturation at 95 °C for 10 min, followed by 45 cycles of 95 °C for 15 s and 60 °C for 60 s. For *Plasmodium*/*Haemoproteus*, thermal cycling conditions included an initial denaturation at 95 °C for 10 min, followed by 45 cycles of 95 °C for 15 s, 57 °C for 45 s, and 72 °C for 30 s. All samples were analyzed in duplicate. Each qPCR plate additionally included negative controls, a standard curve, and an inter-plate calibrator sample (“golden sample”), which was used to correct for inter-plate variation. Inter-plate differences were corrected by comparing Ct values of the calibrator sample among plates. The resulting Ct deviation was used to adjust Ct values of all samples on a given plate prior to conversion to parasite-copy-number estimates. Amplification specificity was verified by inspection of melt curves, and only reactions meeting predefined quality criteria were included in the analyses. Parasite load was estimated from standard curves, corrected using the inter-plate calibrator, and expressed as the mean parasite gene copy number obtained from duplicate reactions. Hereafter, we refer to the calibrated number of parasite gene copies detected per reaction containing 50 ng of total DNA as qPCR-derived parasite load. Because these estimates were not normalized relative to host DNA copy number, they represent relative differences in parasite load among individuals rather than direct estimates of parasitemia.

### 2.3. Statistical Analysis

We used regression modelling, including mixed-effects models where appropriate, to test whether parental blood parasite infections were associated with reproductive performance.

We analyzed reproductive traits at two levels. At the nest level, we considered clutch size, hatching date, and brood size recorded near the end of the nestling period, occurring on day 14 in tits and day 12 in collared flycatchers. At the nestling level, we analyzed body mass on day 8, body mass near fledging age, body mass gain between these two measurements, and nestling body condition to capture both early growth and offspring phenotype near the end of the nestling period. Nestling body condition was estimated as residuals from an ordinary least-squares regression of body mass against tarsus length at the final measurement, calculated separately for each species.

Depending on the level of analysis, we used linear models (LMs) and linear mixed-effects models (LMMs). All response variables, including hatching date, nestling body mass, body mass gain, and body condition, clutch size and brood size, were analyzed using Gaussian error distributions. Preliminary assessment of Poisson models models for clutch size and brood size revealed substantial underdispersion, as indicated by Pearson dispersion statistics and simulation-based residual diagnostics implemented in the DHARMa package (v. 0.5.0) [36]. Because these reproductive traits are bounded count variables with limited ranges, Gaussian models provided a more appropriate representation of the data and were therefore used in all final analyses.

For each reproductive trait, we fitted separate models using either parental infection status or qPCR-derived parasite load as explanatory variables. Infection status was analyzed separately for four parasite categories: (i) combined *Plasmodium*/*Haemoproteus* infection (hereafter “malaria”); (ii) *Plasmodium* infection; (iii) *Haemoproteus* infection; and (iv) *Trypanosoma* infection. Infection status was included as a binary variable indicating whether a given parent was infected or uninfected within the respective parasite category. Models initially included female infection status, male infection status, and their interaction. Full and reduced models were compared using Akaike’s Information Criterion (AIC), and interactions were retained whenever supported by model fit. Reduced models were used only when the simpler model provided an equivalent explanation of the data.

Parasite load was analyzed separately for combined *Plasmodium*/*Haemoproteus* infection (hereafter “malaria”) and for *Trypanosoma* infection. These analyses were restricted to infected individuals and used the calibrated number of parasite gene copies per qPCR reaction as a measure of infection intensity. Because parasite load was available only for infected parents and the number of breeding attempts with both parents infected was limited, female and male parasite loads were analyzed in separate parent-specific models. Parasite load was log-transformed prior to analysis to reduce right-skewness and improve model fit.

Because the three bird species differed in breeding biology, migratory behavior, and sample size, analyses were conducted separately for each species. Year and study plot were included as fixed factors to account for temporal and spatial variation. Although some individual parents were recorded in both study years (15 individuals), only two parental pairs were observed breeding together in both seasons. For nestling-level analyses, nest identity (BOX_ID) was included as a random effect to account for non-independence of nestlings originating from the same nest. For nest-level analyses, breeding attempt was the unit of analysis, and models did not include random effects. Full model structures are provided in Supplementary Materials, Table S3 (infection status models) and Table S4 (infection-intensity models). Because blue tits were represented by substantially smaller sample sizes than the other study species, statistical power was lower for this species. Therefore, blue tit results are not discussed in detail in the main text, and full model outputs are provided in the Supplementary Materials (Tables S5–S8).

All statistical analyses were conducted in R version 4.5.2 [37]. Linear and generalized linear mixed-effects models were fitted using the lme4 package (v. 1.1-38) [38]. Parameter estimates and standard errors were extracted from model coefficients. Predictor significance was evaluated using Type II Wald χ^2^ tests implemented in the car package (v. 3.1-3) [39] via the Anova() function. For models containing female × male interaction terms, coefficient estimates for female and male infection status represent conditional effects evaluated at the reference level of the partner’s infection-status variable. Because a large number of statistical tests were performed across species, parasite groups, parental sexes, and response variables, we controlled for multiple testing using the Benjamini–Hochberg false discovery rate (FDR) procedure [40]. FDR correction was applied separately to the complete set of infection-status models and to the complete set of infection-intensity models, irrespective of whether the analyzed traits were measured at the nest level or nestling level. Thus, all *p*-values obtained from infection-status analyses were adjusted jointly, and all *p*-values obtained from infection-intensity analyses were adjusted jointly. FDR-adjusted *p*-values (*p*_BH_) are reported together with unadjusted *p*-values in the main text and Supplementary Materials.

## 3. Results

### 3.1. Effects of Parental Infection Status on Nestling Traits

**Figure 2:**
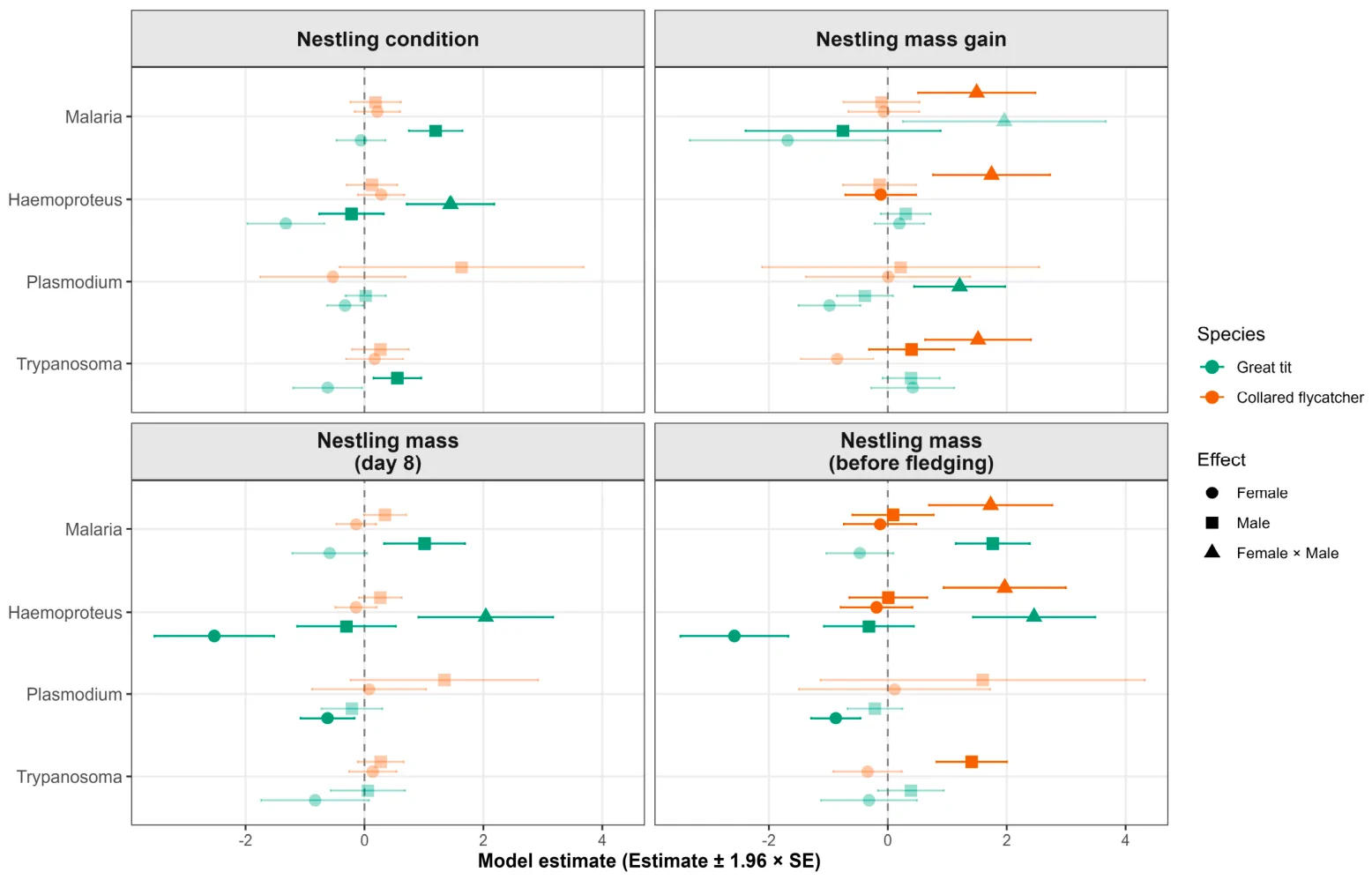
Associations between parental infection status and offspring traits in great tits and collared flycatchers. Points represent model coefficient estimates and horizontal bars represent estimate ± 1.96 × SE. Colors indicate species, whereas symbols denote effects associated with female infection status, male infection status, or the interaction between female and male infection status. Parasite groups are shown on the *y*-axis. Positive estimates indicate higher offspring trait values associated with parental infection, whereas negative estimates indicate lower offspring trait values. All tested associations are shown. More opaque symbols and error bars indicate effects that remained statistically significant after Benjamini–Hochberg false discovery rate correction (FDR; *p*_BH_ < 0.05), whereas semi-transparent symbols and error bars indicate non-significant effects.

Several significant associations between parental infection status and offspring phenotype were detected in great tits and collared flycatchers (Table 2; Figure 2). In great tits, paternal malaria infection was positively associated with nestling condition and body mass at day 8 and body mass before fledging, whereas maternal *Plasmodium* infection was negatively associated with nestling body mass at day 8 and body mass before fledging (Table 2; Figure 2). In addition, significant associations involving *Trypanosoma* infection status were detected, including a positive relationship between paternal infection and nestling condition (Table 2; Figure 2).

**Figure 3:**
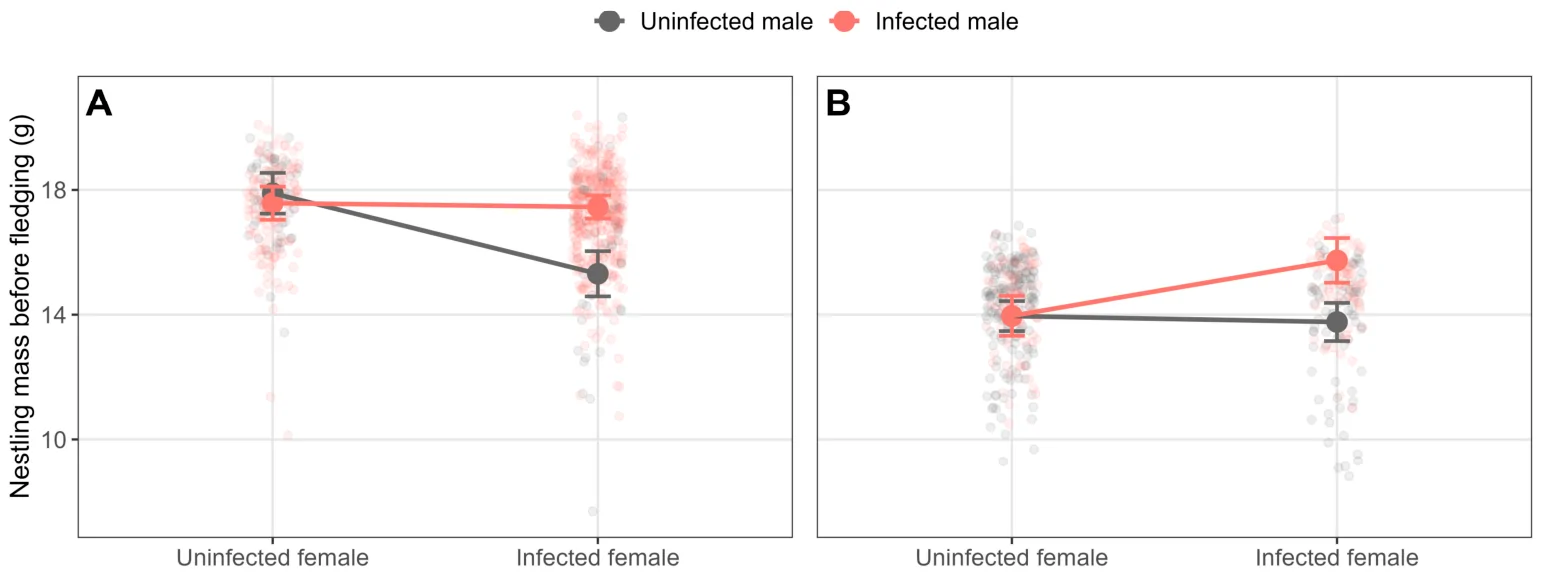
Interaction between female and male *Haemoproteus* infection status in relation to nestling mass before fledging in great tits (**A**) and collared flycatchers (**B**). Large points represent estimated marginal means (EMMs) derived from the fitted models, and error bars indicate 95% confidence intervals. Predicted values are shown for offspring produced by uninfected and infected females paired with uninfected or infected males. Small semi-transparent points represent raw observations.

Several significant female × male interactions were also detected (Table 2, Figure 2). Because the strongest and most consistent effects involved parental *Haemoproteus* infection status, they are presented separately in Section 3.2 (Figure 3). Full results for all interaction models are provided in Supplementary Materials, Table S6.

### 3.2. Female × Male Interactions in Infection Status

Significant interactions between female and male infection status were detected for several offspring traits, with the strongest and most consistent effects involving parental *Haemoproteus* infection status and nestling mass before fledging (Figure 3).

In great tits, the negative association between maternal *Haemoproteus* infection and nestling mass before fledging was evident only when males were uninfected (Figure 3A). In contrast, nestling mass did not differ between infected and uninfected females when males were infected.

A different pattern was observed in collared flycatchers (Figure 3B). When males were uninfected, nestling mass before fledging was similar in offspring produced by infected and uninfected females. However, when males were infected, offspring produced by infected females attained higher body mass than offspring produced by uninfected females (Table 2).

### 3.3. Effects of Parasite Intensity on Offspring Phenotype

**Figure 4:**
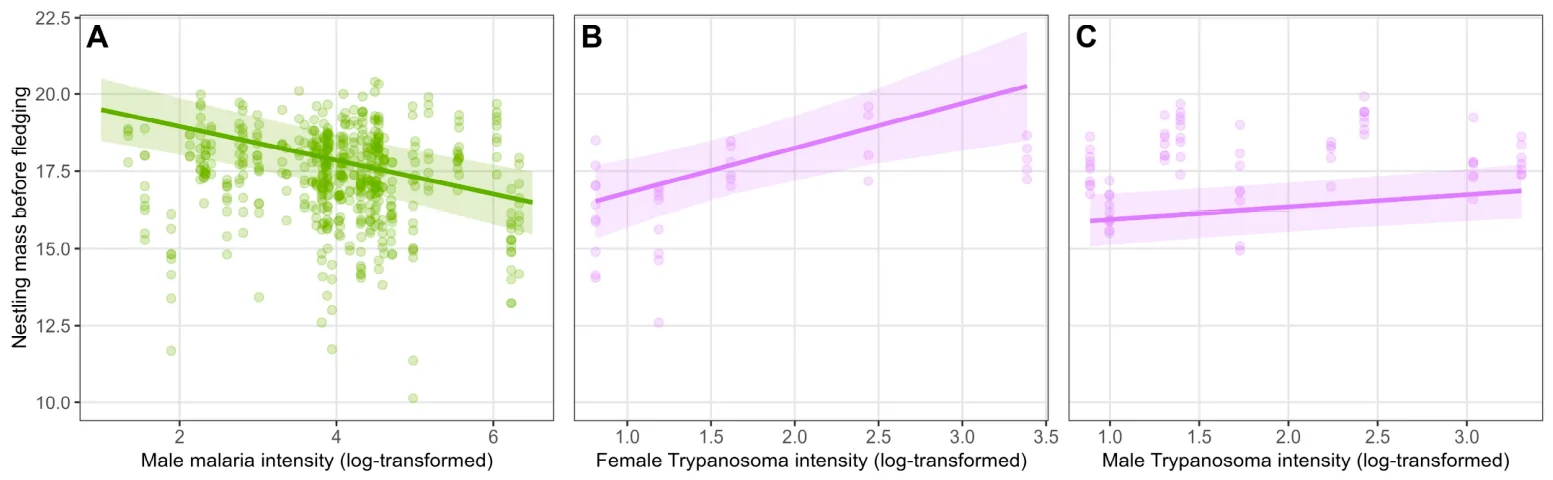
Associations between parental parasite intensity and nestling mass before fledging in great tits. Panel (**A**) shows the relationship between male malaria intensity and nestling mass before fledging. Panels (**B**,**C**) show the relationships between female (**B**) and male (**C**) Trypanosoma intensity and nestling mass before fledging. Lines represent model predictions and shaded areas indicate 95% confidence intervals. Points represent individual observations. Green symbols, lines, and confidence intervals represent malaria intensity analyses, whereas purple symbols, lines, and confidence intervals represent *Trypanosoma* intensity analyses.

Most significant relationships between parental parasite intensity and offspring traits were detected in great tits, whereas few significant associations were observed in collared flycatchers and blue tits (Supplementary Materials, Table S8). The strongest effects, observed in great tits, involved male malaria intensity and *Trypanosoma* intensity in both sexes (Figure 4). Male malaria intensity was negatively associated with nestling body mass before fledging (Figure 4A). In contrast, *Trypanosoma* intensity was positively associated with nestling mass before fledging in both females and males, with a stronger relationship observed for females than for males (Figure 4B,C).

Notably, some associations detected for parasite intensity differed from those observed for infection status. For example, whereas malaria infection status in male great tits was positively associated with offspring condition and body mass (Figure 2; Table 2), malaria intensity showed negative associations with the same traits (Supplementary Table S8). These contrasting patterns indicate that infection status and parasite intensity did not always provide consistent information regarding associations between infection and offspring phenotype.

Additional analyses of reproductive traits (clutch size, brood size before fledging, and hatching date) are presented in Supplementary Tables S5 and S7 for infection status and parasite intensity, respectively. Overall, associations with reproductive traits were less frequent and generally weaker than those detected for offspring traits. Detailed results for blue tits are provided in Supplementary Tables S5–S8 owing to lower sample sizes in this species.

## 4. Discussion

Our results demonstrate that associations between avian blood parasite infections and reproductive performance are highly context-dependent. While relatively few effects were detected for reproductive traits measured at the brood level, numerous associations were found for offspring traits, particularly nestling body mass, growth, and condition. Both positive and negative relationships were observed, indicating that blood parasite infections do not impose uniformly negative reproductive consequences. Instead, infection-related effects depended on parasite taxon, host species, parental sex, and the way infection was characterized. Particularly noteworthy was that infection status and parasite intensity did not always yield consistent results.

The clearest example of contrasting associations between infection status and parasite intensity involved malaria infections in male great tits. Whereas malaria infection status was positively associated with nestling body mass and condition, increasing parasite intensity among infected males showed the opposite pattern. Similar mismatches were observed for other host species, parasite taxon, and reproductive traits. These findings suggest that infection status and parasite intensity are not interchangeable measures of infection but rather reflect different aspects of host–parasite interactions. A similar interpretational issue was highlighted by Westerdahl et al. [41], who argued that analyses based solely on infection prevalence cannot distinguish between hosts that are more susceptible to infection and those that become infected but are able to effectively limit parasite proliferation. They proposed that considering both infection status and infection intensity is necessary to separate host susceptibility from the ability to control parasite development and thereby correctly interpret the biological significance of infection-related patterns. Avian blood parasites frequently persist in their hosts as long-term chronic infections [6], meaning that individuals classified simply as “infected” may differ substantially in parasite burden and the associated physiological costs. Under such conditions, infection status primarily reflects the presence of parasites, whereas parasite intensity may provide additional information about infection severity and its potential physiological costs. Consequently, analyses based exclusively on infection status may obscure biologically meaningful variation among infected individuals. For example, Asghar et al. [14] found that chronic haemosporidian infections were not associated with several host traits when only infection status was considered, whereas analyses incorporating parasitaemia revealed clear infection-related costs. Our interpretation is also consistent with the meta-regression conducted by Knowles et al. [26], who showed that the strength of associations between parasites and life-history traits depends on how infection is characterized, with quantitative measures of parasite burden often revealing stronger associations than infection presence alone. More broadly, studies increasingly emphasize that parasite burden and infection status may capture different components of host–parasite interactions and should not necessarily be treated as equivalent measures of infection [30,42]. This view is reinforced by a recent meta-analysis of avian blood parasites, whose authors identified the scarcity of parasite intensity data as a major limitation of the current literature [22]. The authors further noted that classifying individuals simply as infected or uninfected may oversimplify biological reality and reduce our ability to detect subtle costs of infection. Taken together, these findings suggest that future studies may benefit from incorporating both infection status and parasite intensity whenever possible, as these measures can provide complementary insights into the ecological consequences of parasitic infections.

The heterogeneous associations observed in our study are consistent with growing evidence that the consequences of parasitic infections often vary among host species and between sexes. Most significant relationships were detected in great tits, whereas relatively few effects were observed in collared flycatchers and blue tits. Similar interspecific variation has been reported in previous studies of avian blood parasites and is thought to reflect differences in host ecology, life-history strategy, immune investment, and evolutionary history with parasites [25]. Consequently, even host species breeding in the same environment may differ substantially in their responses to infection and in the reproductive consequences of infection. This may be particularly relevant in the case of resident tits and the migratory collared flycatcher, as migratory hosts are exposed to different parasite communities throughout the annual cycle and may experience infection costs during energetically demanding migratory periods, potentially altering both host–parasite interactions and the reproductive consequences of infection [43,44].

Variation was also evident within species, particularly between females and males. Several associations were sex-specific, with infection affecting different reproductive traits in females and males. Such patterns are consistent with the widespread phenomenon of sex-biased parasitism and growing evidence that the prevalence, intensity, and consequences of infection frequently differ between females and males [27,28]. These differences may arise from sex-specific exposure, immune regulation, resource allocation, and reproductive roles. Infections in females may directly affect reproductive investment and offspring provisioning, whereas infections in males may influence territory defense, mate provisioning, and parental care. Experimental evidence further shows that reducing haemosporidian infection in females can increase parental care, reproductive success, and offspring condition [12], while behavioral differences associated with reproduction may simultaneously generate sex-specific parasite exposure [28]. Similar sex-specific reproductive consequences of avian malaria infections have been reported in other bird species [29].

Beyond sex-specific effects, our results indicate that the reproductive consequences of infection may also depend on the infection status of both parents. Significant interactions between female and male infection status, particularly for *Haemoproteus* infections, indicate that infection consequences cannot always be understood by considering mothers and fathers independently. Similar patterns have been reported previously, with the reproductive consequences of infection depending on the combined infection status of pair members rather than the condition of either parent alone [16,45]. More broadly, sex-specific reproductive roles can generate strong interdependence between partners, such that reproductive investment, parasite exposure, and infection costs experienced by one parent may influence the condition and reproductive performance of the other [31]. Together, these findings indicate that pair-level processes may play an important role in shaping infection–reproduction relationships.

Another notable finding was that several associations between infection and offspring traits were positive rather than negative. Similar patterns have been reported previously in studies of avian haemosporidians, where infected individuals sometimes exhibit equal or even superior reproductive performance compared with uninfected conspecifics [16,30,46]. However, such findings do not imply that parasites are beneficial to their hosts. Instead, infected individuals may represent a non-random subset of the population composed of high-quality hosts that are able to survive, reproduce successfully, and tolerate the costs of infection. Under this scenario, positive associations arise because individuals capable of persisting with infection are of higher intrinsic quality. This interpretation is consistent with the concept of parasite-mediated selection [47]. An alternative, non-mutually exclusive explanation is that some infected individuals may increase current reproductive effort when infection reduces future reproductive prospects, as predicted by the terminal investment hypothesis [48]. Distinguishing between selective survival of high-quality individuals, infection tolerance, and terminal investment would require long-term studies integrating infection history, parasite intensity, survival, and lifetime reproductive success.

Several limitations should be considered when interpreting our results. First, the study was observational and therefore cannot establish causal relationships between infection and reproductive performance. This limitation is particularly important because previous work conducted in the same collared flycatcher population showed that nestlings in poorer condition were more likely to become infected with haemosporidian parasites later in life [49]. Such findings suggest that variation in host condition may influence subsequent infection risk, just as infection may influence host performance, making the direction of causality difficult to resolve in observational studies. In addition, unmeasured host characteristics, including parental age, physiological condition, previous infection history, and other aspects of individual quality, may have contributed to some of the observed associations. Second, haemosporidian infection intensity was quantified jointly for *Plasmodium* and *Haemoproteus*. Consequently, variation in parasite load could not be attributed to either genus individually, despite potentially differing biological effects and virulence. Third, we did not assess *Leucocytozoon* infections. Because *Leucocytozoon* can be highly prevalent in passerine populations [50,51], some birds classified as uninfected in the present study may nevertheless have carried infections by this parasite genus. Consequently, the omission of *Leucocytozoon* may have contributed to unexplained variation in reproductive traits and may have obscured part of the overall relationship between haemosporidian infections and reproductive performance. Fourth, parasite-load estimates were not normalized to host DNA quantity and should therefore be interpreted as relative rather than absolute measures of infection intensity. Variation in the amount and cellular composition of host DNA among blood samples may affect direct comparisons of parasite-load values among individuals. Fifth, although we controlled for multiple testing using the Benjamini–Hochberg false discovery rate procedure, the large number of statistical models evaluated increases the possibility that some weak associations may represent false-positive findings. Consequently, individual associations should be interpreted with appropriate caution, and greater emphasis should be placed on the overall patterns emerging across analyses. Sixth, the study was based on data collected during only two breeding seasons (2019 and 2021). Short-term datasets may capture transient ecological conditions and infection patterns that do not fully reflect longer-term host–parasite dynamics. Seasonal variation in parasite transmission, infection intensity, and resource availability may also influence reproductive performance and could contribute to some of the observed variation among individuals and years. Because these factors were not quantified directly, their contribution could not be assessed in the present study. Finally, some analyses, particularly those involving blue tits and parasite-intensity models, were based on relatively small sample sizes. Consequently, statistical power was limited for some comparisons, and weaker associations may have remained undetected. In addition, nestling condition was estimated using residuals from a regression of body mass on tarsus length. Although widely used in ecological studies, residual-based indices have recognized statistical limitations [52,53,54]. Consequently, results involving nestling condition should be interpreted with appropriate caution. Importantly, nestling condition represented only one of several offspring traits analyzed, and the main conclusions of the study were supported by complementary measures of offspring phenotype, including body mass at day 8, body mass before fledging, and mass gain. Despite these limitations, our results suggest that a more complete understanding of the reproductive consequences of blood parasite infections may be achieved by considering both infection status and infection intensity while accounting for sex-specific effects and the infection status of both parents.

## 5. Conclusions

Blood parasite infections were associated with multiple aspects of reproductive performance in wild birds, but these associations were highly context-dependent. Most significant associations involved offspring traits rather than brood-level reproductive parameters. Multiple robust relationships remained significant after false discovery rate correction, including sex-specific associations and female × male infection-status interactions, particularly for *Haemoproteus* infections.

Our findings demonstrate that the reproductive consequences of blood parasite infections cannot be generalized across host–parasite systems. Moreover, analyses based on infection status and parasite intensity sometimes yielded different conclusions, indicating that these measures provide complementary information about host–parasite interactions. Together, these results highlight the complexity of avian host–parasite interactions and emphasize the importance of accounting for multiple biological sources of variation when evaluating the ecological consequences of blood parasite infections.

Our findings also highlight the importance of considering infection intensity, parental sex, and female × male infection interactions when evaluating the ecological consequences of parasite infections. Future studies should integrate these factors and, where possible, employ longitudinal or experimental approaches to better disentangle causal relationships between parasite infection and host reproductive performance.

## Figures and Tables

**Table 1:** Primer pairs used for qPCR-based quantification of *Plasmodium*/*Haemoproteus* and *Trypanosoma* parasite load and for preparation of standard curve fragments.

Target	Assay	Primer	Sequence (5′–3′)	Product Length
*Plasmodium*/*Haemoproteus*	standard curve	292F	CGGTAGATAGGGAACAAACTGC	335 bp
		631R	GCGAGAAGGGAAGTGTGTTTC	
	qPCR	343F	GCTCACGCATCGCTTCT	188 bp
		496R	GACCGGTCATTTTCTTTG	
*Trypanosoma*	standard curve	T1617F	CTCGATCCCCTGAATGGTGG	456 bp
		T2072R	GCGGTGTGTACAAATGGCAG	
	qPCR	T1871F	GACACGCGCACTACAATGTC	172 bp
		T2042R	GGCACAGTTTGATGAGCTGC	

Primers 292F/631R and 343F/496R were described by Fallon et al. [35]. *Trypanosoma* primers were designed for this study based on avian *Trypanosoma* sequences published by Zídková et al. [8].

**Table 2:** Effects of parental infection status with *Plasmodium*, *Haemoproteus*, and *Trypanosoma* parasites on nestling condition, body mass at day 8, body mass before fledging, and nestling mass gain in great tits and collared flycatchers. Estimates and standard errors are model coefficients obtained from linear mixed-effects models. For parasite types exhibiting significant female × male interactions, estimates are shown for the full model including the interaction term; otherwise, estimates are presented for the reduced model after removal of the interaction term based on model selection using Akaike’s Information Criterion (AIC). χ^2^ statistics and associated *p*-values were obtained from Type II Wald χ^2^ tests. Benjamini–Hochberg false discovery rate-adjusted *p*-values (*p*_BH_ ) are also reported. Only parasite-related effects are presented; year and study plot were included as fixed effects in all models to account for temporal and spatial variation. Full model outputs, including coefficients for year and study plot, are provided in Supplementary Materials Table S6. Significant effects after FDR correction (*p*_BH_ < 0.05) are shown in bold. Dashes (—) indicate that the female × male interaction term was not retained in the final model.

Response Variable	Infection Type	Effect	Great Tit (N_nestlings_ = 631; N_nest_ = 87)						Collared Flycatcher (N_nestlings_ = 415; N_nest_ = 76)					
			Estimate	SE	χ^2^	df	*p*	*p*_BH_	Estimate	SE	χ^2^	df	*p*	*p*_BH_
Nestling condition	malaria	Female	−0.06	0.21	0.08	1	0.778	0.866	0.22	0.20	1.22	1	0.270	0.473
		Male	**1.20**	**0.23**	**27.41**	**1**	**<0.001**	**<0.001**	0.19	0.22	0.73	1	0.392	0.587
	*Haemoproteus*	Female	−1.32	0.33	1.84	1	0.175	0.372	0.28	0.20	1.98	1	0.159	0.349
		Male	**−0.22**	**0.28**	**9.69**	**1**	**0.002**	**0.016**	0.13	0.22	0.34	1	0.559	0.700
		Female × Male	**1.45**	**0.38**	**14.90**	**1**	**<0.001**	**0.002**	—	—	—	—	—	—
	*Plasmodium*	Female	−0.33	0.15	4.52	1	**0.033**	0.114	−0.53	0.62	0.73	1	0.394	0.587
		Male	0.02	0.17	0.01	1	0.907	0.938	1.63	1.05	2.43	1	0.119	0.286
	*Trypanosoma*	Female	−0.62	0.30	4.37	1	**0.037**	0.122	0.17	0.25	0.48	1	0.491	0.643
		Male	**0.55**	**0.20**	**7.37**	**1**	**0.007**	**0.039**	0.27	0.24	1.21	1	0.271	0.473
Nestling mass at day 8	malaria	Female	−0.59	0.32	3.33	1	0.068	0.193	−0.14	0.17	0.65	1	0.419	0.610
		Male	**1.01**	**0.35**	**8.47**	**1**	**0.004**	**0.024**	0.35	0.18	3.58	1	0.059	0.172
	*Haemoproteus*	Female	**−2.53**	**0.51**	**13.13**	**1**	**<0.001**	**0.004**	−0.14	0.18	0.64	1	0.423	0.613
		Male	**−0.30**	**0.42**	**8.22**	**1**	**0.004**	**0.026**	0.27	0.18	2.11	1	0.147	0.330
		Female × Male	**2.04**	**0.58**	**12.48**	**1**	**<0.001**	**0.005**	—	—	—	—	—	0.000
	*Plasmodium*	Female	**−0.62**	**0.23**	**7.21**	**1**	**0.007**	**0.042**	0.08	0.49	0.03	1	0.874	0.932
		Male	−0.21	0.26	0.65	1	0.420	0.610	1.35	0.81	2.80	1	0.095	0.247
	*Trypanosoma*	Female	−0.83	0.46	3.22	1	**0.073**	0.201	0.14	0.20	0.47	1	0.492	0.643
		Male	0.06	0.32	0.03	1	0.856	0.916	0.27	0.20	1.91	1	0.167	0.359
Nestling mass before fledging	malaria	Female	−0.47	0.29	2.67	1	0.102	0.260	**−0.13**	**0.31**	**7.02**	**1**	**0.008**	**0.045**
		Male	**1.77**	**0.32**	**31.00**	**1**	**<0.001**	**<0.001**	**0.09**	**0.35**	**10.35**	**1**	**0.001**	**0.012**
		Female × Male	—	—	—	—	—	—	**1.73**	**0.53**	**10.68**	**1**	**0.001**	**0.011**
	*Haemoproteus*	Female	**−2.58**	**0.46**	**9.04**	**1**	**0.003**	**0.020**	**−0.19**	**0.31**	**7.26**	**1**	**0.007**	**0.041**
		Male	**−0.32**	**0.39**	**15.89**	**1**	**<0.001**	**0.002**	**0.01**	**0.33**	**9.35**	**1**	**0.002**	**0.018**
		Female × Male	**2.46**	**0.52**	**22.03**	**1**	**<0.001**	**<0.001**	**1.97**	**0.52**	**14.09**	**1**	**<0.001**	**0.003**
	*Plasmodium*	Female	**−0.88**	**0.21**	**17.12**	**1**	**<0.001**	**<0.001**	0.11	0.82	0.02	1	0.890	0.936
		Male	−0.22	0.24	0.84	1	0.359	0.566	1.60	1.39	1.31	1	0.252	0.454
	*Trypanosoma*	Female	−0.32	0.41	0.59	1	0.441	0.622	−0.34	0.30	1.32	1	0.251	0.454
		Male	0.39	0.28	1.88	1	0.171	0.365	**1.41**	**0.30**	**21.80**	**1**	**<0.001**	**<0.001**
Nestling mass gain	malaria	Female	−1.68	0.84	0.19	1	0.660	0.785	−0.07	0.30	6.45	1	0.011	0.053
		Male	**−0.75**	**0.84**	**15.75**	**1**	**<0.001**	**0.002**	−0.11	0.33	4.28	1	**0.039**	0.127
		Female × Male	1.96	0.87	5.06	1	**0.024**	0.091	**1.50**	**0.50**	**8.83**	**1**	**0.003**	**0.023**
	*Haemoproteus*	Female	0.19	0.21	0.83	1	0.362	0.566	**−0.12**	**0.30**	**6.99**	**1**	**0.008**	**0.045**
		Male	0.30	0.22	1.96	1	0.161	0.349	−0.14	0.31	4.43	1	**0.035**	0.119
		Female × Male	—	—	—	—	—	—	**1.75**	**0.50**	**12.10**	**1**	**0.001**	**0.006**
	*Plasmodium*	Female	−0.98	0.27	4.12	1	**0.042**	0.136	0.01	0.71	0.00	1	0.993	0.996
		Male	−0.39	0.24	0.02	1	0.891	0.936	0.22	1.19	0.03	1	0.856	0.916
		Female × Male	**1.21**	**0.39**	**9.54**	**1**	**0.002**	**0.017**	—	—	—	—	—	0.000
	*Trypanosoma*	Female	0.42	0.36	1.38	1	0.241	0.447	−0.85	0.31	2.06	1	0.151	0.336
		Male	0.39	0.25	2.53	1	0.112	0.274	**0.40**	**0.37**	**16.87**	**1**	**<0.001**	**<0.001**
		Female × Male	—	—	—	—	—	—	**1.52**	**0.45**	**11.16**	**1**	**0.001**	**0.009**

## Data Availability

The data and R code supporting the findings of this study are publicly available in Zenodo at DOI: https://doi.org/10.5281/zenodo.22665867.

## Supplementary Materials

The following supporting information can be downloaded at https://www.mdpi.com/article/10.3390/biology15181583/s1, Table S1: Sample sizes for infection-status and infection-intensity analyses; Table S2: Haemosporidian parasite lineages detected in the present study; Table S3: Statistical models of parental infection status and reproductive traits; Table S4: Statistical models of parental parasite intensity and reproductive traits; Table S5: Full results of models testing associations between parental infection status and reproductive traits; Table S6: Full results of models testing associations between parental infection status and nestling traits; Table S7: Full results of models testing associations between parental parasite intensity and reproductive traits; Table S8: Full results of models testing associations between parental parasite intensity and nestling traits.
