# Supplementary material for: Blood Parasite Infections and Reproductive Performance in a Multi-Host Avian System
Source: Biology (Basel). 2026 Sep 9;15(18):1583. doi: 10.3390/biology15181583 (PMC13603540; doi:10.3390/biology15181583)
Supplement: Supplementary file 1 [file biology-15-01583-s001.zip › biology-4502685-supplementary.pdf]

Supplementary Information

Blood Parasite Infections and Reproductive Performance in a Multi-Host Avian System

Karolina Żukowska [1], Lars Gustafsson [2], Edyta Podmokła [1, *]

[1] *Department of Comparative Anatomy, Institute of Zoology and Biomedical Research, Faculty of Biology, Jagiellonian University, Gronostajowa 9, 30-387, Kraków, Poland*

[2] *Department of Ecology and Genetics, Evolutionary Biology Centre, Uppsala University, Norbyvägen 18 D, SE-752 36 Uppsala, Sweden*

* *Author for correspondence (edyta.podmokla@uj.edu.pl)*

Table of contents:

Supplementary tables: 2

Table S1. Sample sizes for infection-status and infection-intensity analyses. 2

Table S2. Haemosporidian parasite lineages detected in the present study. 3

Table S3. Statistical models of parental infection status and reproductive traits. 4

Table S4. Statistical models of parental parasite intensity and reproductive traits. 6

Table S5. Full results of models testing associations between parental infection status and reproductive traits. 8

Table S6. Full results of models testing associations between parental infection status and nestling traits. 10

Table S7. Full results of models testing associations between parental parasite intensity and reproductive traits. 14

Table S8. Full results of models testing associations between parental parasite intensity and nestling traits. 16

Table S1. Sample sizes used in infection-status and infection-intensity analyses for great tits (*Parus major*), blue tits (*Cyanistes caeruleus*), and collared flycatchers (*Ficedula albicollis*). For infection-status analyses, $N_{nestlings}$ and N_{nests} indicate the total numbers of nestlings and nests available for a given species, respectively. N_{infF} and N_{infM} denote the numbers of infected females and males. The four female × male infection-combination columns report the numbers of nests in which neither parent was infected (N_{none_inf}), only the female was infected (N_{onlyF_inf}), only the male was infected (N_{onlyM_inf}), or both parents were infected (N_{both_inf}). For infection-intensity analyses, $N_{females}$ and N_{males} indicate the numbers of infected females and males for which qPCR-derived parasite-load estimates were available. $N_{nestlings\_infF}$ and $N_{nestlings\_infM}$ represent the numbers of nestling observations associated with those females and males. Some parasite-intensity models could not be estimated because of limited sample size or insufficient variation; exact sample sizes used in individual analyses are reported in Tables S7 and S8.

Species	Infection type	Infection status				female × male infection combinations				Infection intensity			
		$N_{nestlings}$	N_{nests}	N_{infF}	N_{infM}	N_{none_inf}	N_{onlyF_inf}	N_{onlyM_inf}	N_{both_inf}	$N_{females}$	N_{males}	$N_{nestlings\_infF}$	$N_{nestlings\_infM}$
**Great tit**	malaria	631	87	72	78	2	7	13	65	66	73	477	529
	Haemoproteus			60	68	10	9	17	51				
	Plasmodium			36	43	28	16	23	20				
	Trypanosoma			6	12	70	5	11	1	5	9	33	71
**Blue tit**	malaria	155	18	14	10	3	5	1	9	14	10	128	92
	Haemoproteus			10	8	7	3	1	7				
	Plasmodium			7	2	11	5	0	2				
	Trypanosoma			6	3	10	5	2	1	5	3	37	24
**Collared flycatcher**	malaria	415	76	30	28	32	16	14	14	22	19	127	103
	Haemoproteus			27	27	34	15	15	12				
	Plasmodium			3	1	72	3	1	0				
	Trypanosoma			21	22	41	13	14	8	18	19	107	113

Table S2. Haemosporidian parasite lineages detected in the adult birds included in the present study. Numbers indicate infected individuals carrying a given lineage. Percentages represent the overall prevalence of *Haemoproteus* and *Plasmodium* infections within each host species.

	**Bird species:**	**Great tit**	**Blue tit**	**Collared flycatcher**
**Species**	**Lineage**	N = 174	N = 36	N = 152
Haemoproteus		**128 (73.6%)**	**18 (50.0%)**	**54 (35.5%)**
belopolskyi	ARW1	5		3
pallidus	COLL2			4
balmorali	COLL3	33	10	26
majoris	PARUS1	72	11	
majoris	PHSIB1	88	1	23
	RW2	4		7
majoris	WW2	11		
Plasmodium		**79 (45.4%)**	**9 (25.0%)**	**4 (2.6%)**
circumflexum	BT7	20	2	
	COLL7			1
	GRW09			1
relictum	GRW11	6		
	LAMPUR03			1
	RTSR1			1
relictum	SGS1	13	2	
polare	SW2	34		
circumflexum	TURDUS1	22	5	

Table S3. Structure of statistical models testing associations between parental infection status and reproductive traits. Nestling-level models were fitted as linear mixed-effects models with nest-box identity (BOX_ID) included as a random intercept. Nest-level models did not include random effects. Interactions between maternal and paternal infection status were initially included in all models and subsequently removed when the reduced model was supported by model selection based on ΔAIC ≤ 2.

	Response variable	Infection type	Model specification	Distribution	Function
**Nest-level models**	**Clutch size**	malaria	CS ~ PARENT_F_MALARIA * PARENT_M_MALARIA + YEAR + PLOT	Gaussian	lm
		Haemoproteus	CS ~ PARENT_F_HAEMO * PARENT_M_HAEMO + YEAR + PLOT	Gaussian	lm
		Plasmodium	CS ~ PARENT_F_PLASMO * PARENT_M_PLASMO + YEAR + PLOT	Gaussian	lm
		Trypanosoma	CS ~ PARENT_F_TRYP * PARENT_M_TRYP + YEAR + PLOT	Gaussian	lm
	**Brood size before fledging**	malaria	BSL ~ PARENT_F_MALARIA * PARENT_M_MALARIA + YEAR + PLOT	Gaussian	lm
		Haemoproteus	BSL ~ PARENT_F_HAEMO * PARENT_M_HAEMO + YEAR + PLOT	Gaussian	lm
		Plasmodium	BSL ~ PARENT_F_PLASMO * PARENT_M_PLASMO + YEAR + PLOT	Gaussian	lm
		Trypanosoma	BSL ~ PARENT_F_TRYP * PARENT_M_TRYP + YEAR + PLOT	Gaussian	lm
	**Hatching date**	malaria	OH ~ PARENT_F_MALARIA * PARENT_M_MALARIA + YEAR + PLOT	Gaussian	lm
		Haemoproteus	OH ~ PARENT_F_HAEMO * PARENT_M_HAEMO + YEAR + PLOT	Gaussian	lm
		Plasmodium	OH ~ PARENT_F_PLASMO * PARENT_M_PLASMO + YEAR + PLOT	Gaussian	lm
		Trypanosoma	OH ~ PARENT_F_TRYP * PARENT_M_TRYP + YEAR + PLOT	Gaussian	lm

	Response variable	Infection type	Model specification	Distribution	Function
**Nestling-level models**	**Nestling condition**	malaria	CONDITION ~ PARENT_F_MALARIA * PARENT_M_MALARIA + YEAR + PLOT + (1 | BOX_ID)	Gaussian	lmer
		Haemoproteus	CONDITION ~ PARENT_F_HAEMO * PARENT_M_HAEMO + YEAR + PLOT + (1 | BOX_ID)	Gaussian	lmer
		Plasmodium	CONDITION ~ PARENT_F_PLASMO * PARENT_M_PLASMO + YEAR + PLOT + (1 | BOX_ID)	Gaussian	lmer
		Trypanosoma	CONDITION ~ PARENT_F_TRYP * PARENT_M_TRYP + YEAR + PLOT + (1 | BOX_ID)	Gaussian	lmer
	**Nestling mass gain**	malaria	MASS_GAIN ~ PARENT_F_MALARIA * PARENT_M_MALARIA + YEAR + PLOT + (1 | BOX_ID)	Gaussian	lmer
		Haemoproteus	MASS_GAIN ~ PARENT_F_HAEMO * PARENT_M_HAEMO + YEAR + PLOT + (1 | BOX_ID)	Gaussian	lmer
		Plasmodium	MASS_GAIN ~ PARENT_F_PLASMO * PARENT_M_PLASMO + YEAR + PLOT + (1 | BOX_ID)	Gaussian	lmer
		Trypanosoma	MASS_GAIN ~ PARENT_F_TRYP * PARENT_M_TRYP + YEAR + PLOT + (1 | BOX_ID)	Gaussian	lmer
	**Nestling mass at day 8**	malaria	MASS_8 ~ PARENT_F_MALARIA * PARENT_M_MALARIA + YEAR + PLOT + (1 | BOX_ID)	Gaussian	lmer
		Haemoproteus	MASS_8 ~ PARENT_F_HAEMO * PARENT_M_HAEMO + YEAR + PLOT + (1 | BOX_ID)	Gaussian	lmer
		Plasmodium	MASS_8 ~ PARENT_F_PLASMO * PARENT_M_PLASMO + YEAR + PLOT + (1 | BOX_ID)	Gaussian	lmer
		Trypanosoma	MASS_8 ~ PARENT_F_TRYP * PARENT_M_TRYP + YEAR + PLOT + (1 | BOX_ID)	Gaussian	lmer
	**Nestling mass before fledging**	malaria	MASS_L ~ PARENT_F_MALARIA * PARENT_M_MALARIA + YEAR + PLOT + (1 | BOX_ID)	Gaussian	lmer
		Haemoproteus	MASS_L ~ PARENT_F_HAEMO * PARENT_M_HAEMO + YEAR + PLOT + (1 | BOX_ID)	Gaussian	lmer
		Plasmodium	MASS_L ~ PARENT_F_PLASMO * PARENT_M_PLASMO + YEAR + PLOT + (1 | BOX_ID)	Gaussian	lmer
		Trypanosoma	MASS_L ~ PARENT_F_TRYP * PARENT_M_TRYP + YEAR + PLOT + (1 | BOX_ID)	Gaussian	lmer

Table S4. Structure of statistical models testing associations between parasite intensity and reproductive traits. Parasite intensity models were fitted separately for maternal and paternal infections and included only infected individuals. Nestling-level models included nest-box identity (BOX_ID) as a random intercept.

	Response variable	Intensity predictor	Model specification	Distribution	Function
**Nest-level models**	**Clutch size**	Maternal malaria intensity	CS ~ log_F_malaria + YEAR + PLOT	Gaussian	lm
		Paternal malaria intensity	CS ~ log_M_malaria + YEAR + PLOT	Gaussian	lm
		Maternal *Trypanosoma* intensity	CS ~ log_F_tryp + YEAR + PLOT	Gaussian	lm
		Paternal *Trypanosoma* intensity	CS ~ log_M_tryp + YEAR + PLOT	Gaussian	lm
	**Brood size before fledging**	Maternal malaria intensity	BSL ~ log_F_malaria + YEAR + PLOT	Gaussian	lm
		Paternal malaria intensity	BSL ~ log_M_malaria + YEAR + PLOT	Gaussian	lm
		Maternal *Trypanosoma* intensity	BSL ~ log_F_tryp + YEAR + PLOT	Gaussian	lm
		Paternal *Trypanosoma* intensity	BSL ~ log_M_tryp + YEAR + PLOT	Gaussian	lm
	**Hatching date**	Maternal malaria intensity	OH ~ log_F_malaria + YEAR + PLOT	Gaussian	lm
		Paternal malaria intensity	OH ~ log_M_malaria + YEAR + PLOT	Gaussian	lm
		Maternal *Trypanosoma* intensity	OH ~ log_F_tryp + YEAR + PLOT	Gaussian	lm
		Paternal *Trypanosoma* intensity	OH ~ log_M_tryp + YEAR + PLOT	Gaussian	lm

	Response variable	Intensity predictor	Model specification	Distribution	Function
**Nestling-level models**	**Nestling condition**	Maternal malaria intensity	CONDITION ~ log_F_malaria + YEAR + PLOT + (1 | BOX_ID)	Gaussian	lmer
		Paternal malaria intensity	CONDITION ~ log_M_malaria + YEAR + PLOT + (1 | BOX_ID)	Gaussian	lmer
		Maternal *Trypanosoma* intensity	CONDITION ~ log_F_tryp + YEAR + PLOT + (1 | BOX_ID)	Gaussian	lmer
		Paternal *Trypanosoma* intensity	CONDITION ~ log_M_tryp + YEAR + PLOT + (1 | BOX_ID)	Gaussian	lmer
	**Nestling mass gain**	Maternal malaria intensity	MASS_GAIN ~ log_F_malaria + YEAR + PLOT + (1 | BOX_ID)	Gaussian	lmer
		Paternal malaria intensity	MASS_GAIN ~ log_M_malaria + YEAR + PLOT + (1 | BOX_ID)	Gaussian	lmer
		Maternal *Trypanosoma* intensity	MASS_GAIN ~ log_F_tryp + YEAR + PLOT + (1 | BOX_ID)	Gaussian	lmer
		Paternal *Trypanosoma* intensity	MASS_GAIN ~ log_M_tryp + YEAR + PLOT + (1 | BOX_ID)	Gaussian	lmer
	**Nestling mass at day 8**	Maternal malaria intensity	MASS_8 ~ log_F_malaria + YEAR + PLOT + (1 | BOX_ID)	Gaussian	lmer
		Paternal malaria intensity	MASS_8 ~ log_M_malaria + YEAR + PLOT + (1 | BOX_ID)	Gaussian	lmer
		Maternal *Trypanosoma* intensity	MASS_8 ~ log_F_tryp + YEAR + PLOT + (1 | BOX_ID)	Gaussian	lmer
		Paternal *Trypanosoma* intensity	MASS_8 ~ log_M_tryp + YEAR + PLOT + (1 | BOX_ID)	Gaussian	lmer
	**Nestling mass before fledging**	Maternal malaria intensity	MASS_L ~ log_F_malaria + YEAR + PLOT + (1 | BOX_ID)	Gaussian	lmer
		Paternal malaria intensity	MASS_L ~ log_M_malaria + YEAR + PLOT + (1 | BOX_ID)	Gaussian	lmer
		Maternal *Trypanosoma* intensity	MASS_L ~ log_F_tryp + YEAR + PLOT + (1 | BOX_ID)	Gaussian	lmer
		Paternal *Trypanosoma* intensity	MASS_L ~ log_M_tryp + YEAR + PLOT + (1 | BOX_ID)	Gaussian	lmer

Table S5. Full results of models testing associations between parental infection status and reproductive traits in great tits (*Parus major*), blue tits (*Cyanistes caeruleus*), and collared flycatchers (*Ficedula albicollis*). Response variables included clutch size, brood size before fledging, and hatching date. Estimates and standard errors are model coefficients obtained from linear models. For parasite types exhibiting significant female × male interactions, estimates are shown for the full model including the interaction term; otherwise, estimates are presented for reduced models after removal of the interaction term based on model selection using Akaike's Information Criterion (AIC). Test statistics were obtained from Type II ANOVA tests. Benjamini-Hochberg false discovery rate-adjusted P-values (p_{BH}) are also reported. Dashes (—) indicate that the female × male interaction term was not retained in the final model. Effects remaining significant after FDR correction ($p_{BH} < 0.05$) are shown in bold.

Response variable	Infection type	Effect	Great tit (*Parus major*) (N = 87)						Blue tit (*Cyanistes caeruleus*) (N = 18)						Collared flycatcher (*Ficedula albicollis*) (N = 76)					
			Estimate	SE	Test statistic	df	*p*	p_{BH}	Estimate	SE	Test statistic	df	*p*	p_{BH}	Estimate	SE	Test statistic	df	*p*	p_{BH}
**Clutch size**	malaria	Female	0.16	0.39	0.17	1	0.681	0.804	**-1.99**	**0.62**	**10.41**	**1**	**0.009**	**0.047**	0.25	0.18	1.82	1	0.182	0.381
		Male	-0.31	0.50	0.39	1	0.536	0.685	-0.83	0.61	1.88	1	0.200	0.402	-0.02	0.19	0.01	1	0.912	0.938
		YEAR	—	—	**6.93**	**1**	**0.010**	**0.051**	—	—	**35.66**	**1**	**<0.001**	**0.002**	—	—	0.54	1	0.466	0.639
		PLOT	—	—	1.40	4	0.242	0.447	—	—	3.35	4	**0.055**	0.164	—	—	2.55	4	**0.047**	0.148
	Haemoproteus	Female	0.23	0.33	0.50	1	0.482	0.643	**-2.59**	**0.73**	**12.54**	**1**	**0.005**	**0.162**	0.31	0.19	2.75	1	0.102	0.260
		Male	-0.03	0.38	0.01	1	0.929	0.948	-0.79	0.75	1.12	1	0.315	0.785	-0.06	0.19	0.08	1	0.773	0.864
		YEAR	—	—	5.36	1	**0.023**	0.089	—	—	**36.17**	**1**	**<0.001**	**0.011**	—	—	0.53	1	0.467	0.639
		PLOT	—	—	1.36	4	0.254	0.454	—	—	2.57	4	0.103	0.301	—	—	2.68	4	**0.039**	0.127
	Plasmodium	Female	0.30	0.34	0.78	1	0.381	0.578	-0.78	0.72	1.19	1	0.301	0.606	-0.22	0.44	0.25	1	0.619	0.757
		Male	0.19	0.34	0.33	1	0.569	0.710	-0.90	1.10	0.66	1	0.435	0.785	0.59	0.76	0.61	1	0.440	0.622
		YEAR	—	—	6.78	1	**0.011**	0.053	—	—	7.78	1	**0.019**	0.072	—	—	0.45	1	0.506	0.654
		PLOT	—	—	0.88	4	0.478	0.643	—	—	1.12	4	0.401	0.643	—	—	2.07	4	0.094	0.247
	Trypanosoma	Female	-0.06	0.58	0.01	1	0.913	0.938	0.10	0.80	0.02	1	0.905	0.938	0.19	0.20	0.84	1	0.361	0.566
		Male	-0.08	0.42	0.03	1	0.856	0.916	1.40	1.14	1.50	1	0.249	0.454	-0.04	0.20	0.04	1	0.846	0.916
		YEAR	—	—	6.41	1	**0.013**	0.060	—	—	**8.86**	**1**	**0.014**	**0.061**	—	—	0.70	1	0.407	0.601
		PLOT	—	—	1.37	4	0.253	0.454	—	—	1.21	4	0.366	0.569	—	—	2.19	4	0.079	0.212
**Brood size before fledging**	malaria	Female	-0.47	0.47	1.00	1	0.321	0.534	4.30	1.63	7.01	1	**0.024**	0.091	0.04	0.31	0.02	1	0.890	0.936
		Male	-0.76	0.61	1.57	1	0.214	0.418	-1.21	1.59	0.57	1	0.466	0.639	-0.32	0.32	0.98	1	0.325	0.537
		YEAR	—	—	5.60	1	**0.020**	0.080	—	—	1.47	1	0.254	0.454	—	—	6.52	1	0.013	0.059
		PLOT	—	—	0.54	4	0.707	0.824	—	—	1.29	4	0.337	0.554	—	—	3.10	4	**0.021**	0.081
	Haemoproteus	Female	-0.36	0.40	0.78	1	0.381	0.578	4.17	2.07	4.07	1	0.071	0.397	0.28	0.32	0.81	1	0.372	0.571

Response variable	Infection type	Effect	Great tit (*Parus major*) (N = 87)						Blue tit (*Cyanistes caeruleus*) (N = 18)						Collared flycatcher (*Ficedula albicollis*) (N = 76)					
			Estimate	SE	Test statistic	df	p	p_{BH}	Estimate	SE	Test statistic	df	p	p_{BH}	Estimate	SE	Test statistic	df	p	p_{BH}
		Male	-0.54	0.46	1.37	1	0.245	0.450	-1.90	2.11	0.82	1	0.388	0.563	-0.38	0.32	1.47	1	0.230	0.435
		YEAR	—	—	**7.98**	**1**	**0.006**	**0.037**	—	—	1.87	1	0.202	0.639	—	—	6.69	1	**0.012**	0.056
		PLOT	—	—	0.61	4	0.656	0.785	—	—	0.66	4	0.631	0.694	—	—	3.12	4	**0.020**	0.080
	Plasmodium	Female	-0.29	0.42	0.48	1	0.490	0.643	0.98	1.59	0.38	1	0.552	0.627	-1.31	0.72	3.27	1	0.075	0.203
		Male	0.05	0.42	0.01	1	0.914	0.938	1.22	2.44	0.25	1	0.629	0.916	0.64	1.25	0.26	1	0.612	0.754
		YEAR	—	—	2.80	1	0.098	0.254	—	—	0.03	1	0.857	0.901	—	—	5.86	1	**0.018**	0.075
		PLOT	—	—	0.35	4	0.847	0.916	—	—	0.50	4	0.738	0.863	—	—	3.25	4	**0.017**	0.071
	Trypanosoma	Female	**-1.98**	**0.67**	**8.71**	**1**	**0.004**	**0.026**	0.46	1.77	0.07	1	0.803	0.885	0.17	0.34	0.24	1	0.623	0.758
		Male	0.71	0.48	2.14	1	0.148	0.330	0.78	2.53	0.10	1	0.764	0.864	0.35	0.32	1.17	1	0.284	0.484
		YEAR	—	—	**6.95**	**1**	**0.010**	**0.051**	—	—	0.01	1	0.925	0.946	—	—	4.72	1	**0.033**	0.114
		PLOT	—	—	0.87	4	0.488	0.643	—	—	0.48	4	0.751	0.855	—	—	2.30	4	0.067	0.193
**Hatching date**	malaria	Female	-2.02	1.33	2.31	1	0.132	0.301	-0.01	2.07	0.00	1	0.998	0.998	0.22	0.80	0.07	1	0.790	0.875
		Male	0.57	1.70	0.11	1	0.740	0.849	-0.61	2.03	0.09	1	0.769	0.864	-0.77	0.83	0.86	1	0.358	0.566
		YEAR	—	—	**13.14**	**1**	**0.001**	**0.006**	—	—	**14.37**	**1**	**0.004**	**0.024**	—	—	**25.14**	**1**	**<0.001**	**<0.001**
		PLOT	—	—	1.47	4	0.218	0.420	—	—	0.62	4	0.658	0.785	—	—	0.07	4	0.992	0.996
	Haemoproteus	Female	**-3.36**	**1.07**	**9.79**	**1**	**0.003**	**0.020**	-0.10	2.30	0.00	1	0.965	0.864	0.13	0.83	0.02	1	0.879	0.935
		Male	-0.63	1.23	0.26	1	0.613	0.754	-2.37	2.34	1.02	1	0.336	0.618	-0.55	0.83	0.43	1	0.513	0.661
		YEAR	—	—	**7.36**	**1**	**0.008**	**0.045**	—	—	7.04	1	**0.024**	0.068	—	—	24.37	1	**<0.001**	**<0.001**
		PLOT	—	—	1.86	4	0.126	0.297	—	—	0.68	4	0.623	0.785	—	—	0.09	4	0.984	0.993
	Plasmodium	Female	-1.27	1.16	1.20	1	0.277	0.482	-1.15	1.54	0.55	1	0.474	0.700	0.21	1.92	0.01	1	0.913	0.938
		Male	1.03	1.16	0.79	1	0.378	0.577	1.62	2.36	0.47	1	0.507	0.618	-3.35	3.31	1.03	1	0.315	0.529
		YEAR	—	—	**7.66**	**1**	**0.007**	**0.041**	—	—	**20.85**	**1**	**0.001**	**0.011**	—	—	23.48	1	**<0.001**	**<0.001**
		PLOT	—	—	1.42	4	0.236	0.441	—	—	0.68	4	0.624	0.759	—	—	0.13	4	0.970	0.981
	Trypanosoma	Female	-3.07	1.92	2.56	1	0.114	0.276	1.89	1.70	0.21	1	0.661	0.785	0.43	0.86	0.26	1	0.615	0.754
		Male	2.73	1.39	3.86	1	0.053	0.161	4.12	2.46	1.08	1	0.325	0.537	-1.87	0.82	5.20	1	**0.026**	0.095
		YEAR	—	—	**11.51**	**1**	**0.001**	**0.011**	—	—	**15.62**	**1**	**0.003**	**0.024**	—	—	**26.72**	**1**	**<0.001**	**<0.001**
		PLOT	—	—	1.93	4	0.114	0.276	—	—	0.79	4	0.559	0.700	—	—	0.18	4	0.947	0.963

Table S6. Full results of models testing associations between parental infection status and nestling traits in great tits (*Parus major*), blue tits (*Cyanistes caeruleus*), and collared flycatchers (*Ficedula albicollis*). Response variables included nestling condition, body mass at day 8, body mass before fledging, and nestling mass gain. Estimates and standard errors are model coefficients obtained from linear mixed-effects models. For parasite types exhibiting significant female × male interactions, estimates are shown for the full model including the interaction term; otherwise, estimates are presented for reduced models after removal of the interaction term based on model selection using Akaike's Information Criterion (AIC). Test statistics were obtained from Type II Wald χ^2 tests. Benjamini-Hochberg false discovery rate-adjusted P-values (p_{BH}) are also reported. Dashes (—) indicate that the female × male interaction term was not retained in the final model. Effects remaining significant after FDR correction (p_{BH} < 0.05) are shown in bold.

Response variable	Infection type	Effect	Great tit (*Parus major*) (Nnestlings = 631; Nnest = 87)						Blue tit (Cyanistes caeruleus) (Nnestlings = 155; Nnest = 18)						Collared flycatcher (Ficedula albicollis) (Nnestlings = 415; Nnest = 76)					
			Estimate	SE	χ^2	df	p	p_{BH}	Estimate	SE	χ^2	df	p	p_{BH}	Estimate	SE	χ^2	df	p	p_{BH}
**Nestling condition**	**malaria**	Female	-0.06	0.21	0.08	1	0.778	0.866	0.22	0.26	5.09	1	**0.024**	0.091	0.22	0.20	1.22	1	0.270	0.473
		Male	**1.20**	**0.23**	**27.41**	**1**	**<0.001**	**<0.001**	**-1.66**	**0.50**	**6.87**	**1**	**0.009**	**0.047**	0.19	0.22	0.73	1	0.392	0.587
		Female × Male	—	—	—	—	—	—	1.28	0.53	5.87	1	**0.015**	0.067	—	—	—	—	—	—
		YEAR	—	—	**18.16**	**1**	**<0.001**	**<0.001**	—	—	**18.43**	**1**	**<0.001**	**<0.001**	—	—	1.60	1	0.206	0.410
		PLOT	—	—	5.70	4	0.223	0.425	—	—	**25.19**	**4**	**<0.001**	**<0.001**	—	—	5.03	4	0.284	0.484
	Haemoproteus	Female	-1.32	0.33	1.84	1	0.175	0.372	0.12	0.27	1.38	1	0.240	0.154	0.28	0.20	1.98	1	0.159	0.349
		Male	**-0.22**	**0.28**	**9.69**	**1**	**0.002**	**0.016**	**-1.84**	**0.48**	**7.71**	**1**	**0.006**	**0.012**	0.13	0.22	0.34	1	0.559	0.700
		Female × Male	**1.45**	**0.38**	**14.90**	**1**	**<0.001**	**0.002**	**1.34**	**0.49**	**7.48**	**1**	**0.006**	**0.059**	—	—	—	—	—	—
		YEAR	—	—	**12.33**	**1**	**<0.001**	**0.005**	—	—	**10.23**	**1**	**0.001**	**0.003**	—	—	1.97	1	0.161	0.349
		PLOT	—	—	3.69	4	0.450	0.627	—	—	**24.96**	**4**	**<0.001**	**<0.001**	—	—	4.78	4	0.310	0.524
	Plasmodium	Female	-0.33	0.15	4.52	1	0.033	0.114	0.40	0.22	3.35	1	0.067	0.178	-0.53	0.62	0.73	1	0.394	0.587
		Male	0.02	0.17	0.01	1	0.907	0.938	-0.02	0.33	0.00	1	0.964	0.643	1.63	1.05	2.43	1	0.119	0.286
		Female × Male	—	—	—	—	—	—	—	—	—	—	—	—	—	—	—	—	—	—
		YEAR	—	—	**11.84**	**1**	**0.001**	**0.007**	—	—	**8.83**	**1**	**0.003**	**0.015**	—	—	1.41	1	0.236	0.441
		PLOT	—	—	0.61	4	0.962	0.976	—	—	7.72	4	0.102	0.381	—	—	6.19	4	0.186	0.384
	Trypanosoma	Female	-0.62	0.30	4.37	1	0.037	0.122	0.02	0.17	0.02	1	0.896	0.938	0.17	0.25	0.48	1	0.491	0.643
		Male	**0.55**	**0.20**	**7.37**	**1**	**0.007**	**0.039**	**1.19**	**0.24**	**24.31**	**1**	**<0.001**	**<0.001**	0.27	0.24	1.21	1	0.271	0.473
		Female × Male	—	—	—	—	—	—	—	—	—	—	—	—	—	—	—	—	—	—
		YEAR	—	—	**9.40**	**1**	**0.002**	**0.018**	—	—	**8.76**	**1**	**0.003**	**0.023**	—	—	2.67	1	0.102	0.260

Response variable	Infection type	Effect	Great tit (*Parus major*) (Nnestlings = 631; Nnest = 87)						Blue tit (Cyanistes caeruleus) (Nnestlings = 155; Nnest = 18)						Collared flycatcher (Ficedula albicollis) (Nnestlings = 415; Nnest = 76)					
			Estimate	SE	χ^2	df	*p*	p_{BH}	Estimate	SE	χ^2	df	*p*	p_{BH}	Estimate	SE	χ^2	df	*p*	p_{BH}
		PLOT	—	—	1.80	4	0.772	0.864	—	—	**23.14**	**4**	**<0.001**	**0.002**	—	—	5.03	4	0.284	0.484
**Nestling mass at day 8**	**malaria**	Female	-0.59	0.32	3.33	1	0.068	0.193	0.95	0.47	4.09	1	**0.043**	0.138	-0.14	0.17	0.65	1	0.419	0.610
		Male	**1.01**	**0.35**	**8.47**	**1**	**0.004**	**0.024**	-0.85	0.46	3.52	1	0.061	0.177	0.35	0.18	3.58	1	0.059	0.172
		Female × Male	—	—	—	—	—	—	—	—	—	—	—	—	—	—	—	—	—	—
		YEAR	—	—	4.70	1	0.030	0.107	—	—	4.88	1	**0.027**	0.099	—	—	2.38	1	0.123	0.292
		PLOT	—	—	6.05	4	0.196	0.397	—	—	4.73	4	0.316	0.529	—	—	4.45	4	0.348	0.562
	Haemoproteus	Female	**-2.53**	**0.51**	**13.13**	**1**	**0.000**	**0.004**	1.16	0.54	4.63	1	**0.031**	0.051	-0.14	0.18	0.64	1	0.423	0.613
		Male	**-0.30**	**0.42**	**8.22**	**1**	**0.004**	**0.026**	-0.96	0.54	3.09	1	**0.079**	0.078	0.27	0.18	2.11	1	0.147	0.330
		Female × Male	**2.04**	**0.58**	**12.48**	**1**	**<0.001**	**0.005**	—	—	—	—	—	—	—	—	—	—	—	—
		YEAR	—	—	0.45	1	0.502	0.654	—	—	**6.08**	**1**	**0.014**	**0.053**	—	—	2.36	1	0.124	0.294
		PLOT	—	—	7.60	4	0.107	0.267	—	—	3.47	4	0.483	0.402	—	—	3.77	4	0.438	0.622
	Plasmodium	Female	**-0.62**	**0.23**	**7.21**	**1**	**0.007**	**0.042**	0.42	0.40	1.08	1	0.300	0.461	0.08	0.49	0.03	1	0.874	0.932
		Male	-0.21	0.26	0.65	1	0.420	0.610	-0.21	0.61	0.12	1	0.728	0.643	1.35	0.81	2.80	1	0.095	0.247
		Female × Male	—	—	—	—	—	—	—	—	—	—	—	—	—	—	—	—	—	—
		YEAR	—	—	**7.83**	**1**	**0.005**	**0.032**	—	—	3.53	1	0.060	0.149	—	—	2.53	1	0.112	0.274
		PLOT	—	—	1.57	4	0.813	0.891	—	—	2.16	4	0.706	0.875	—	—	4.05	4	0.400	0.593
	Trypanosoma	Female	-0.83	0.46	3.22	1	0.073	0.201	0.85	0.39	4.66	1	**0.031**	0.108	0.14	0.20	0.47	1	0.492	0.643
		Male	0.06	0.32	0.03	1	0.856	0.916	0.69	0.56	1.53	1	0.216	0.418	0.27	0.20	1.91	1	0.167	0.359
		Female × Male	—	—	—	—	—	—	—	—	—	—	—	—	—	—	—	—	—	—
		YEAR	—	—	3.19	1	0.074	0.203	—	—	0.89	1	0.344	0.560	—	—	0.88	1	0.349	0.562
		PLOT	—	—	3.48	4	0.482	0.643	—	—	2.23	4	0.694	0.814	—	—	3.92	4	0.418	0.610
**Nestling mass before fledging**	**malaria**	Female	-0.47	0.29	2.67	1	0.102	0.260	0.15	0.36	2.63	1	0.105	0.262	**-0.13**	**0.31**	**7.02**	**1**	**0.008**	**0.045**
		Male	1.77	0.32	31.00	1	<0.001	0.000	-1.73	0.69	1.57	1	0.210	0.415	**0.09**	**0.35**	**10.35**	**1**	**0.001**	**0.012**
		Female × Male	—	—	—	—	—	—	1.58	0.73	4.75	1	0.029	0.106	**1.73**	**0.53**	**10.68**	**1**	**0.001**	**0.011**
		YEAR	—	—	**11.05**	**1**	**0.001**	**0.010**	—	—	**8.25**	**1**	**0.004**	**0.026**	—	—	0.13	1	0.716	0.829
		PLOT	—	—	9.25	4	0.055	0.164	—	—	10.65	4	**0.031**	0.108	—	—	6.13	4	0.190	0.390

Response variable	Infection type	Effect	Great tit (*Parus major*) (Nnestlings = 631; Nnest = 87)						Blue tit (Cyanistes caeruleus) (Nnestlings = 155; Nnest = 18)						Collared flycatcher (Ficedula albicollis) (Nnestlings = 415; Nnest = 76)					
			Estimate	SE	χ^2	df	*p*	p_{BH}	Estimate	SE	χ^2	df	*p*	p_{BH}	Estimate	SE	χ^2	df	*p*	p_{BH}
	Haemoproteus	Female	**-2.58**	**0.46**	**9.04**	**1**	**0.003**	**0.020**	0.12	0.38	0.95	1	0.330	0.218	**-0.19**	**0.31**	**7.26**	**1**	**0.007**	**0.041**
		Male	**-0.32**	**0.39**	**15.89**	**1**	**0.000**	**0.002**	-1.85	0.67	2.08	1	0.149	0.197	**0.01**	**0.33**	**9.35**	**1**	**0.002**	**0.018**
		Female × Male	**2.46**	**0.52**	**22.03**	**1**	**<0.001**	**<0.001**	1.61	0.68	5.61	1	**0.018**	0.000	**1.97**	**0.52**	**14.09**	**1**	**<0.001**	**0.003**
		YEAR	—	—	4.48	1	0.034	0.116	—	—	4.97	1	**0.026**	0.153	—	—	0.02	1	0.891	0.936
		PLOT	—	—	9.89	4	0.042	0.136	—	—	10.48	4	**0.033**	0.209	—	—	5.65	4	0.227	0.431
	Plasmodium	Female	**-0.88**	**0.21**	**17.12**	**1**	**<0.001**	**<0.001**	0.46	0.27	2.95	1	0.086	0.418	0.11	0.82	0.02	1	0.890	0.936
		Male	-0.22	0.24	0.84	1	0.359	0.566	0.07	0.41	0.03	1	0.859	0.885	1.60	1.39	1.31	1	0.252	0.454
		Female × Male	—	—	—	—	—	—	—	—	—	—	—	—	—	—	—	—	—	—
		YEAR	—	—	**15.79**	**1**	**<0.001**	**0.002**	—	—	2.88	1	0.090	0.193	—	—	5.39	1	**0.020**	0.080
		PLOT	—	—	3.74	4	0.442	0.622	—	—	4.58	4	0.334	0.643	—	—	6.65	4	0.156	0.343
	Trypanosoma	Female	-0.32	0.41	0.59	1	0.441	0.622	0.08	0.24	0.12	1	0.729	0.838	-0.34	0.30	1.32	1	0.251	0.454
		Male	0.39	0.28	1.88	1	0.171	0.365	**1.25**	**0.34**	**13.31**	**1**	**<0.001**	**0.004**	**1.41**	**0.30**	**21.80**	**1**	**<0.001**	**<0.001**
		Female × Male	—	—	—	—	—	—	—	—	—	—	—	—	—	—	—	—	—	—
		YEAR	—	—	**6.82**	**1**	**0.009**	**0.047**	—	—	1.50	1	0.222	0.425	—	—	1.17	1	0.280	0.484
		PLOT	—	—	6.30	4	0.178	0.376	—	—	12.57	4	**0.014**	0.061	—	—	4.40	4	0.355	0.564
**Nestling mass gain**	**malaria**	Female	-1.68	0.84	0.19	1	0.660	0.785	-0.99	0.38	1.75	1	0.186	0.384	**-0.07**	**0.30**	**6.45**	**1**	**0.011**	**0.053**
		Male	**-0.75**	**0.84**	**15.75**	**1**	**<0.001**	**0.002**	-1.58	0.73	2.13	1	0.144	0.327	-0.11	0.33	4.28	1	0.039	0.127
		Female × Male	1.96	0.87	5.06	1	**0.024**	0.091	**2.41**	**0.77**	**9.90**	**1**	**0.002**	**0.014**	**1.50**	**0.50**	**8.83**	**1**	**0.003**	**0.023**
		YEAR	—	—	0.91	1	0.341	0.557	—	—	0.06	1	0.815	0.891	—	—	0.73	1	0.393	0.587
		PLOT	—	—	2.13	4	0.711	0.826	—	—	3.05	4	0.550	0.696	—	—	3.43	4	0.489	0.643
	Haemoproteus	Female	0.19	0.21	0.83	1	0.362	0.566	-1.09	0.40	4.40	1	**0.036**	0.262	**-0.12**	**0.30**	**6.99**	**1**	**0.008**	**0.045**
		Male	0.30	0.22	1.96	1	0.161	0.349	-1.24	0.70	1.19	1	0.275	0.411	-0.14	0.31	4.43	1	0.035	0.119
		Female × Male	—	—	—	—	—	—	**2.02**	**0.71**	**8.14**	**1**	**0.004**	**0.026**	**1.75**	**0.50**	**12.10**	**1**	**0.001**	**0.006**
		YEAR	—	—	2.27	1	0.132	0.301	—	—	1.14	1	0.286	0.816	—	—	1.12	1	0.291	0.493
		PLOT	—	—	4.26	4	0.371	0.571	—	—	5.25	4	0.262	0.571	—	—	3.23	4	0.520	0.668
	Plasmodium	Female	-0.98	0.27	4.12	1	0.042	0.136	0.04	0.33	0.02	1	0.902	0.852	0.01	0.71	0.00	1	0.993	0.996

Response variable	Infection type	Effect	Great tit (*Parus major*) (Nnestlings = 631; Nnest = 87)						Blue tit (Cyanistes caeruleus) (Nnestlings = 155; Nnest = 18)						Collared flycatcher (Ficedula albicollis) (Nnestlings = 415; Nnest = 76)					
			Estimate	SE	χ^2	df	*p*	p_{BH}	Estimate	SE	χ^2	df	*p*	p_{BH}	Estimate	SE	χ^2	df	*p*	p_{BH}
		Male	-0.39	0.24	0.02	1	0.891	0.936	0.29	0.50	0.33	1	0.564	0.654	0.22	1.19	0.03	1	0.856	0.916
		Female × Male	**1.21**	**0.39**	**9.54**	**1**	**0.002**	**0.017**	—	—	—	—	—	—	—	—	—	—	—	—
		YEAR	—	—	0.27	1	0.603	0.747	—	—	0.80	1	0.370	0.587	—	—	0.36	1	0.550	0.696
		PLOT	—	—	3.76	4	0.439	0.622	—	—	1.00	4	0.909	0.938	—	—	5.20	4	0.267	0.471
	Trypanosoma	Female	0.42	0.36	1.38	1	0.241	0.447	**-1.02**	**0.29**	**8.54**	**1**	**0.004**	**0.024**	-0.85	0.31	2.06	1	0.151	0.336
		Male	0.39	0.25	2.53	1	0.112	0.274	0.18	0.41	2.29	1	0.131	0.301	**0.40**	**0.37**	**16.87**	**1**	**<0.001**	**<0.001**
		Female × Male	—	—	—	—	—	—	1.72	0.74	5.39	1	**0.020**	0.080	**1.52**	**0.45**	**11.16**	**1**	**0.001**	**0.009**
		YEAR	—	—	0.16	1	0.689	0.811	—	—	0.12	1	0.725	0.836	—	—	0.87	1	0.352	0.563
		PLOT	—	—	3.58	4	0.465	0.639	—	—	8.69	4	0.070	0.195	—	—	2.76	4	0.599	0.745

Table S7. Full results of models testing associations between parental parasite intensity and reproductive traits in great tits (*Parus major*), blue tits (*Cyanistes caeruleus*), and collared flycatchers (*Ficedula albicollis*). Response variables included clutch size, brood size before fledging, and hatching date. Models were fitted separately for maternal and paternal parasite intensity and included only infected individuals. Estimates and standard errors are model coefficients obtained from the final models. Test statistics were obtained from Type II ANOVA tests. Benjamini-Hochberg false discovery rate-adjusted P-values (p_{BH}) are also reported. N denotes the number of nests included in the analysis. Dashes (—) indicate parameters that could not be estimated because of insufficient variation or limited sample size. Effects remaining significant after FDR correction ($p_{BH} < 0.05$) are shown in bold.

Response variable	Infection type	Effect	Great tit (*Parus major*)							Blue tit (*Cyanistes caeruleus*)							Collared flycatcher (*Ficedula albicollis*)						
			Estimate	SE	χ^2	df	*p*	p_{BH}	N	Estimate	SE	χ^2	df	*p*	p_{BH}	N	Estimate	SE	χ^2	df	*p*	p_{BH}	N
Clutch size	Maternal malaria intensity	Intensity	0.14	0.14	0.98	1	0.326	0.693	66	-0.22	0.24	0.79	1	0.403	0.737	14	0.06	0.08	0.61	1	0.446	0.795	22
		YEAR	—	—	5.08	1	**0.028**	0.155	66	—	—	7.34	1	**0.030**	0.163	14	—	—	0.51	1	0.485	0.826	22
		PLOT	—	—	1.09	4	0.369	0.715	66	—	—	2.33	4	0.155	0.481	14	—	—	0.53	3	0.669	0.922	22
	Paternal malaria intensity	Intensity	0.25	0.16	2.50	1	0.119	0.398	73	0.09	0.22	0.17	1	0.709	0.933	10	0.10	0.11	0.83	1	0.378	0.715	19
		YEAR	—	—	2.31	1	0.133	0.430	73	—	—	2.49	1	0.212	0.584	10	—	—	0.01	1	0.934	0.984	19
		PLOT	—	—	1.55	4	0.198	0.579	73	—	—	2.11	4	0.283	0.651	10	—	—	0.41	3	0.746	0.941	19
	Maternal Trypanosoma intensity	Intensity	—	—	—	—	—	—	—	—	—	—	—	—	—	—	-0.30	0.26	1.33	1	0.271	0.651	18
		YEAR	—	—	—	—	—	—	—	—	—	—	—	—	—	—	—	—	0.37	1	0.557	0.892	18
		PLOT	—	—	—	—	—	—	—	—	—	—	—	—	—	—	—	—	0.09	3	0.962	0.993	18
	Paternal Trypanosoma intensity	Intensity	-1.17	0.72	2.63	1	0.246	0.633	9	—	—	—	—	—	—	—	0.38	0.36	1.07	1	0.321	0.692	18
		YEAR	—	—	0.00	1	0.957	0.993	9	—	—	—	—	—	—	—	—	—	1.03	1	0.330	0.695	18
		PLOT	—	—	0.65	4	0.680	0.922	9	—	—	—	—	—	—	—	—	—	0.26	3	0.853	0.967	18
Brood size before fledging	Maternal malaria intensity	Intensity	0.09	0.18	0.24	1	0.627	0.922	66	0.74	0.64	1.36	1	0.282	0.651	14	-0.05	0.12	0.18	1	0.677	0.922	22
		YEAR	—	—	1.60	1	0.211	0.584	66	—	—	0.12	1	0.743	0.941	14	—	—	1.36	1	0.261	0.638	22
		PLOT	—	—	0.26	4	0.902	0.977	66	—	—	0.43	4	0.784	0.941	14	—	—	0.08	3	0.971	0.993	22
	Paternal malaria intensity	Intensity	0.10	0.20	0.26	1	0.615	0.912	73	0.73	0.65	1.27	1	0.341	0.698	10	0.09	0.13	0.50	1	0.492	0.826	19
		YEAR	—	—	5.73	1	**0.020**	0.117	73	—	—	0.09	1	0.790	0.941	10	—	—	1.95	1	0.186	0.571	19
		PLOT	—	—	0.69	4	0.599	0.905	73	—	—	0.25	4	0.891	0.977	10	—	—	0.39	3	0.763	0.941	19
	Maternal Trypanosoma intensity	Intensity	—	—	—	—	—	—	—	—	—	—	—	—	—	—	-0.37	0.43	0.77	1	0.397	0.731	18
		YEAR	—	—	—	—	—	—	—	—	—	—	—	—	—	—	—	—	1.45	1	0.252	0.633	18
		PLOT	—	—	—	—	—	—	—	—	—	—	—	—	—	—	—	—	0.21	3	0.888	0.977	18
	Paternal Trypanosoma intensity	Intensity	-0.88	0.67	1.71	1	0.321	0.692	9	—	—	—	—	—	—	—	0.47	0.48	0.96	1	0.347	0.698	18
		YEAR	—	—	0.01	1	0.928	0.984	9	—	—	—	—	—	—	—	—	—	0.32	1	0.585	0.896	18
		PLOT	—	—	0.92	4	0.580	0.896	9	—	—	—	—	—	—	—	—	—	0.42	3	0.742	0.941	18

Response variable	Infection type	Effect	Great tit (*Parus major*)							Blue tit (*Cyanistes caeruleus*)							Collared flycatcher (*Ficedula albicollis*)						
			**Estimate**	**SE**	χ^2	**df**	***p***	p_{BH}	**N**	**Estimate**	**SE**	χ^2	**df**	***p***	p_{BH}	**N**	**Estimate**	**SE**	χ^2	**df**	***p***	p_{BH}	**N**
**Hatching date**	**Maternal malaria intensity**	Intensity	-0.20	0.44	0.21	1	0.650	0.922	66	0.16	0.89	0.03	1	0.863	0.968	14	0.00	0.48	0.00	1	0.995	0.995	22
		YEAR	—	—	**14.49**	**1**	**<0.001**	**0.005**	**66**	—	—	3.26	1	0.114	0.387	14	—	—	1.73	1	0.207	0.584	22
		PLOT	—	—	0.61	4	0.660	0.922	66	—	—	0.35	4	0.837	0.964	14	—	—	0.87	3	0.476	0.826	22
	**Paternal malaria intensity**	Intensity	0.22	0.56	0.15	1	0.699	0.933	73	0.64	0.81	0.63	1	0.485	0.826	10	0.02	0.25	0.01	1	0.934	0.984	19
		YEAR	—	—	**8.49**	**1**	**0.005**	**0.047**	**73**	—	—	2.66	1	0.202	0.579	10	—	—	**29.40**	**1**	**<0.001**	**0.002**	**19**
		PLOT	—	—	1.46	4	0.225	0.593	73	—	—	0.33	4	0.842	0.964	10	—	—	0.57	3	0.644	0.922	19
	**Maternal Trypanosoma intensity**	Intensity	—	—	—	—	—	—	—	—	—	—	—	—	—	—	-0.95	0.95	0.99	1	0.340	0.698	18
		YEAR	—	—	—	—	—	—	—	—	—	—	—	—	—	—	—	—	7.87	1	**0.016**	0.117	18
		PLOT	—	—	—	—	—	—	—	—	—	—	—	—	—	—	—	—	0.28	3	0.837	0.964	18
	**Paternal Trypanosoma intensity**	Intensity	3.49	0.38	83.68	1	**0.012**	0.089	9	—	—	—	—	—	—	—	0.25	0.78	0.10	1	0.753	0.941	18
		YEAR	—	—	49.74	1	**0.020**	0.117	9	—	—	—	—	—	—	—	—	—	**43.93**	**1**	**<0.001**	**<0.001**	**18**
		PLOT	—	—	8.39	4	0.109	0.381	9	—	—	—	—	—	—	—	—	—	1.09	3	0.390	0.725	18

Table S8. Full results of models testing associations between parental parasite intensity and nestling traits in great tits (*Parus major*), blue tits (*Cyanistes caeruleus*), and collared flycatchers (*Ficedula albicollis*). Response variables included nestling condition, body mass at day 8, body mass before fledging, and nestling mass gain. Models were fitted separately for maternal and paternal parasite intensity and included only infected individuals. Estimates and standard errors are model coefficients obtained from the final models. Test statistics were obtained from Type II Wald χ^2 tests. Benjamini-Hochberg false discovery rate-adjusted P-values (p_{BH}) are also reported. N denotes the number of nestling observations. Dashes (—) indicate parameters that could not be estimated because of insufficient variation or limited sample size. Effects remaining significant after FDR correction (p_{BH} < 0.05) are shown in bold.

Response variable	Infection type	Effect	Great tit (*Parus major*)							Blue tit (*Cyanistes caeruleus*)							Collared flycatcher (*Ficedula albicollis*)						
			Est.	SE	χ^2	df	*p*	p_{BH}	N	Est.	SE	χ^2	df	*p*	p_{BH}	N	Est.	SE	χ^2	df	*p*	p_{BH}	N
**Nestling condition**	**Maternal malaria intensity**	Intensity	0.00	0.08	0.00	1	0.988	0.995	477	-0.05	0.09	0.29	1	0.588	0.896	128	-0.01	0.14	0.00	1	0.957	0.993	127
		YEAR	—	—	**16.04**	**1**	**<0.001**	**0.002**	**477**	—	—	**16.13**	**1**	**<0.001**	**0.002**	**128**	—	—	1.55	1	0.214	0.584	127
		PLOT	—	—	2.33	4	0.675	0.922	477	—	—	**18.96**	**4**	**0.001**	**0.011**	**128**	—	—	1.09	3	0.779	0.941	127
	**Paternal malaria intensity**	Intensity	-0.16	0.08	4.16	1	**0.041**	0.194	529	0.11	0.05	4.51	1	0.034	0.174	92	0.00	0.11	0.00	1	0.988	0.995	103
		YEAR	—	—	2.84	1	0.092	0.348	529	—	—	2.55	1	0.110	0.381	92	—	—	4.30	1	**0.038**	0.183	103
		PLOT	—	—	1.76	4	0.780	0.941	529	—	—	**35.22**	**4**	**<0.001**	**<0.001**	**92**	—	—	1.85	3	0.605	0.905	103
	**Maternal Trypanosoma intensity**	Intensity	0.32	0.36	0.77	1	0.379	0.715	33	0.48	0.30	2.63	1	0.105	0.380	37	0.35	0.37	0.88	1	0.349	0.698	107
		YEAR	—	—	0.31	1	0.575	0.896	33	—	—	7.11	1	**0.008**	0.069	37	—	—	0.57	1	0.451	0.797	107
		PLOT	—	—	2.76	2	0.252	0.633	33	—	—	4.83	2	0.089	0.348	37	—	—	2.32	3	0.509	0.845	107
	**Paternal Trypanosoma intensity**	Intensity	0.34	0.13	7.06	1	**0.008**	0.069	71	0.14	0.52	0.08	1	0.784	0.941	24	0.10	0.43	0.05	1	0.822	0.964	107
		YEAR	—	—	0.66	1	0.415	0.746	71	—	—	2.85	1	0.091	0.348	24	—	—	0.27	1	0.606	0.905	107
		PLOT	—	—	**64.08**	**4**	**<0.001**	**<0.001**	**71**	—	—	—	0	—	—	24	—	—	1.26	3	0.740	0.941	107
**Nestling mass at day 8**	**Maternal malaria intensity**	Intensity	-0.07	0.12	0.36	1	0.548	0.884	477	-0.04	0.21	0.04	1	0.841	0.964	128	0.06	0.12	0.30	1	0.584	0.896	127
		YEAR	—	—	1.04	1	0.307	0.686	477	—	—	0.07	1	0.792	0.941	128	—	—	0.01	1	0.922	0.984	127
		PLOT	—	—	2.27	4	0.686	0.925	477	—	—	2.04	4	0.729	0.941	128	—	—	0.27	3	0.965	0.993	127
	**Paternal malaria intensity**	Intensity	**-0.34**	**0.12**	**8.61**	**1**	**0.003**	**0.033**	**529**	0.09	0.13	0.51	1	0.476	0.826	92	0.00	0.10	0.00	1	0.995	0.995	103
		YEAR	—	—	3.15	1	0.076	0.310	529	—	—	0.47	1	0.493	0.826	92	—	—	0.32	1	0.571	0.896	103
		PLOT	—	—	4.23	4	0.376	0.715	529	—	—	1.74	4	0.784	0.941	92	—	—	2.87	3	0.413	0.746	103
	**Maternal Trypanosoma intensity**	Intensity	0.65	0.44	2.11	1	0.147	0.463	33	-0.66	0.50	1.71	1	0.191	0.579	37	-0.08	0.28	0.08	1	0.778	0.941	107
		YEAR	—	—	2.25	1	0.134	0.430	33	—	—	0.22	1	0.638	0.922	37	—	—	0.02	1	0.901	0.977	107
		PLOT	—	—	2.98	2	0.225	0.593	33	—	—	4.18	2	0.124	0.408	37	—	—	1.64	3	0.651	0.922	107
	**Paternal Trypanosoma intensity**	Intensity	0.41	0.16	6.61	1	**0.010**	0.083	71	-1.18	1.03	1.30	1	0.255	0.633	24	-0.29	0.27	1.11	1	0.292	0.665	107
		YEAR	—	—	0.01	1	0.906	0.977	71	—	—	**12.81**	**1**	**<0.001**	**0.005**	**24**	—	—	3.39	1	0.066	0.274	107
		PLOT	—	—	76.82	4	0.000	0.000	71	—	—	—	0	—	—	24	—	—	**16.86**	**3**	**0.001**	**0.011**	**107**

Response variable	Infection type	Effect	Great tit (*Parus major*)							Blue tit (*Cyanistes caeruleus*)							Collared flycatcher (*Ficedula albicollis*)						
			Est.	SE	χ^2	df	*p*	p_{BH}	N	Est.	SE	χ^2	df	*p*	p_{BH}	N	Est.	SE	χ^2	df	*p*	p_{BH}	N
**Nestling mass before fledging**	**Maternal malaria intensity**	Intensity	-0.10	0.11	0.86	1	0.354	0.698	477	-0.13	0.13	0.98	1	0.322	0.692	128	0.08	0.22	0.14	1	0.709	0.933	127
		YEAR	—	—	3.49	1	0.062	0.263	477	—	—	2.82	1	0.093	0.348	128	—	—	0.18	1	0.674	0.922	127
		PLOT	—	—	4.41	4	0.354	0.698	477	—	—	5.06	4	0.281	0.651	128	—	—	0.42	3	0.935	0.984	127
	**Paternal malaria intensity**	Intensity	**-0.55**	**0.11**	**26.79**	**1**	**<0.001**	**<0.001**	**529**	0.18	0.08	5.53	1	**0.019**	0.117	92	0.04	0.13	0.07	1	0.791	0.941	103
		YEAR	—	—	1.25	1	0.263	0.638	529	—	—	2.83	1	0.093	0.348	92	—	—	1.53	1	0.217	0.584	103
		PLOT	—	—	3.28	4	0.513	0.846	529	—	—	**22.75**	**4**	**<0.001**	**0.002**	**92**	—	—	3.22	3	0.359	0.702	103
	**Maternal Trypanosoma intensity**	Intensity	**1.45**	**0.41**	**12.41**	**1**	**<0.001**	**0.006**	**33**	0.07	0.35	0.04	1	0.842	0.964	37	1.18	0.57	4.34	1	**0.037**	0.183	107
		YEAR	—	—	3.58	1	0.059	0.259	33	—	—	2.58	1	0.109	0.381	37	—	—	0.03	1	0.854	0.967	107
		PLOT	—	—	6.60	2	**0.037**	0.183	33	—	—	6.08	2	0.048	0.221	37	—	—	0.60	3	0.896	0.977	107
	**Paternal Trypanosoma intensity**	Intensity	**0.39**	**0.13**	**8.78**	**1**	**0.003**	**0.032**	**71**	0.55	0.63	0.77	1	0.382	0.715	24	-0.20	0.34	0.34	1	0.563	0.895	107
		YEAR	—	—	5.54	1	0.019	0.117	71	—	—	1.32	1	0.250	0.633	24	—	—	1.18	1	0.278	0.651	107
		PLOT	—	—	**68.71**	**4**	**<0.001**	**<0.001**	**71**	—	—	—	0	—	—	24	—	—	11.16	3	**0.011**	0.086	107
**Nestling mass gain**	**Maternal malaria intensity**	Intensity	-0.04	0.09	0.23	1	0.636	0.922	477	-0.09	0.12	0.48	1	0.489	0.826	128	0.02	0.15	0.02	1	0.888	0.977	127
		YEAR	—	—	0.87	1	0.351	0.698	477	—	—	1.68	1	0.196	0.579	128	—	—	0.36	1	0.548	0.884	127
		PLOT	—	—	1.86	4	0.763	0.941	477	—	—	11.85	4	**0.019**	0.117	128	—	—	1.69	3	0.639	0.922	127
	**Paternal malaria intensity**	Intensity	-0.16	0.09	3.53	1	0.060	0.261	529	0.09	0.07	1.65	1	0.199	0.579	92	0.04	0.09	0.14	1	0.704	0.933	103
		YEAR	—	—	1.00	1	0.316	0.692	529	—	—	**10.92**	**1**	**0.001**	**0.012**	**92**	—	—	5.58	1	0.018	0.117	103
		PLOT	—	—	3.06	4	0.548	0.884	529	—	—	**18.28**	**4**	**0.001**	**0.013**	**92**	—	—	9.16	3	**0.027**	0.155	103
	**Maternal Trypanosoma intensity**	Intensity	**0.81**	**0.26**	**9.86**	**1**	**0.002**	**0.019**	**33**	0.73	0.34	4.53	1	0.033	0.174	37	1.19	0.45	7.02	1	**0.008**	0.069	107
		YEAR	—	—	0.20	1	0.656	0.922	33	—	—	5.50	1	**0.019**	0.117	37	—	—	0.05	1	0.827	0.964	107
		PLOT	—	—	**30.03**	**2**	**<0.001**	**<0.001**	**33**	—	—	7.51	2	**0.024**	0.137	37	—	—	1.07	3	0.784	0.941	107
	**Paternal Trypanosoma intensity**	Intensity	-0.03	0.20	0.03	1	0.868	0.968	71	1.72	0.90	3.65	1	0.056	0.253	24	0.01	0.43	0.00	1	0.981	0.995	107
		YEAR	—	—	2.63	1	0.105	0.380	71	—	—	**10.82**	**1**	**0.001**	**0.012**	**24**	—	—	0.00	1	0.968	0.993	107
		PLOT	—	—	4.85	4	0.303	0.683	71	—	—	—	0	—	—	24	—	—	0.77	3	0.858	0.967	107
